# D-PRISM: a global survey-based study to assess diagnostic and treatment approaches in pneumonia managed in intensive care

**DOI:** 10.1186/s13054-024-05180-y

**Published:** 2024-11-22

**Authors:** Luis Felipe Reyes, Cristian C. Serrano-Mayorga, Zhongheng Zhang, Isabela Tsuji, Gennaro De Pascale, Valeria Enciso Prieto, Mervyn Mer, Elyce Sheehan, Prashant Nasa, Goran Zangana, Kostoula Arvaniti, Alexis Tabah, Gentle Sunder Shrestha, Hendrik Bracht, Arie Zainul Fatoni, Khalid Abidi, Helmi bin Sulaiman, Vandana Kalwaje Eshwara, Liesbet De Bus, Yoshiro Hayashi, Pervin Korkmaz, Ali Ait Hssain, Niccolò Buetti, Qing Yuan Goh, Arthur Kwizera, Despoina Koulenti, Nathan D. Nielsen, Pedro Povoa, Otavio Ranzani, Jordi Rello, Andrew Conway Morris

**Affiliations:** 1 Unisabana Center for Translational Science, School of Medicine, Universidad de La Sabana, Chia, Colombia; 2 Clinica Universidad de La Sabana, Chia, Colombia; 3 PhD Biosciences Program, Engineering School, Universidad de La Sabana, Chia, Colombia; 4 Pandemic Sciences Institute, University of Oxford, Oxford, UK; 5 Department of Emergency Medicine, Sir Run Run Shaw Hospital, Zhejiang University School of Medicine, Hangzhou, China; 6 Hospital Israelita Albert Einstein, São Paulo, Brazil; 7 Dipartimento di Scienze Biotecnologiche di Base, Cliniche Intensivologiche e Perioperatorie, Università Cattolica del Sacro Cuore, Rome, Italy; 8 Dipartimento di Scienze dell’Emergenza, Anestesiologiche e della Rianimazione, Fondazione Policlinico Universitario A. Gemelli IRCCS, Rome, Italy; 9 Divisions of Critical Care and Pulmonology, Department of Medicine, Charlotte Maxeke Johannesburg Academic Hospital and Faculty of Health Sciences, University of the Witwatersrand, Johannesburg, South Africa; 10 Division of Pulmonary, Critical Care and Sleep Medicine, University of New Mexico School of Medicine, Albuquerque, USA; 11 Critical Care Medicine NMC Specialty Hospital Dubai, Dubai, UAE; 12 Internal Medicine, College of Medicine and Health Sciences, Al Ain, UAE; 13 Department of Acute and General Medicine, Royal Infirmary of Edinburgh, Edinburgh, Scotland, UK; 14 Intensive Care Medicine, Papageorgiou Hospital, Thessaloníki, Greece; 15 Queensland University of Technology, Brisbane, QLD Australia; 16 Intensive Care Unit, Redcliffe Hospital, Metro North Hospital and Health Services, Brisbane, QLD Australia; 17 Faculty of Medicine, The University of Queensland, Brisbane, QLD Australia; 18 Department of Critical Care Medicine, Tribhuvan University Teaching Hospital, Maharajgunj, Kathmandu, Nepal; 19 Department of Anesthesiology, Intensive Care, Emergency Medicine, Transfusion Medicine, and Pain Therapy, Protestant Hospital of the Bethel Foundation, University Hospital of Bielefeld, Campus Bielefeld-Bethel, Bielefeld, Germany; 20 Department of Anesthesiology and Intensive Therapy, Saiful Anwar General Hospital - Faculty of Medicine, Brawijaya University, Malang, East Java Indonesia; 21 Ibn Sina University Hospital, Faculty of Medicine and Pharmacy, Mohammed V University, Rabat, Morocco; 22 Infectious Diseases Unit, Department of Medicine, Faculty of Medicine, University of Malaya, Kuala Lumpur, Malaysia; 23 Department of Microbiology Kasturba Medical College, Manipal Manipal Academy of Higher Education, Manipal, Karnataka India; 24 Department of Intensive Care Medicine, Ghent University Hospital, Ghent, Belgium; 25 Department of Internal Medicine and Pediatrics, Faculty of Medicine and Health Sciences, Ghent University, Ghent, Belgium; 26 Department of Intensive Care Medicine, Kameda Medical Center, Kamogawa, Japan; 27 Pulmonary Disease Department, Ege University School of Medicine, Izmir, Turkey; 28 Medical Intensive Care Unit, Hamad General Hospital, Doha, Qatar; 29 Infection Control Program, Geneva University Hospitals and Faculty of Medicine, World Health Organization Collaborating Centre, Geneva, Switzerland; 30 IAME UMR 1137, INSERM, Université Paris-Cité, Paris, France; 31 Division of Anaesthesiology and Perioperative Medicine, Department of Surgical Intensive Care, Singapore General Hospital, Singapore, Singapore; 32 Department of Anaesthesia, Makerere University, Kampala, Uganda; 33 Department of Critical Care, King’s College Hospital NHS Foundation Trust, London, UK; 34 Antibiotic Optimisation Group, UQ Centre for Clinical Research, Faculty of Medicine, The University of Queensland, Brisbane, Australia; 35 Section of Transfusion Medicine and Therapeutic Pathology, University of New Mexico School of Medicine, Albuquerque, USA; 36 Faculdade de Ciências Médicas, NOVA Medical School, NOVA University of Lisbon, Lisbon, Portugal; 37 Center for Clinical Epidemiology and Research Unit of Clinical Epidemiology, OUH Odense University Hospital, Odense, Denmark; 38 Department of Intensive Care, Hospital de São Francisco Xavier, ULSLO, Lisbon, Portugal; 39 Barcelona Institute for Global Health, ISGlobal, Hospital Clinic-Universitat de Barcelona, Barcelona, Spain; 40 Pulmonary Division, Heart Institute (InCor), Hospital das Clinicas HCFMUSP, Faculdade de Medicina, Universidade de Sao Paulo, São Paulo, Brazil; 41 Vall d’Hebron Institute of Research, Barcelona, Spain; 42 Pormation, Recherche & Évaluation (FOREVA), CHU Nîmes, Nîmes, France; 43 Centro de Investigación Biomédica en Red (CIBERES), Instituto de Salud Carlos III, Madrid, Spain; 44 Division of Perioperative, Acute, Critical Care and Emergency Medicine, Department of Medicine, University of Cambridge, Level 4, Addenbrooke’s Hospital, Hills Road, Cambridge, UK; 45 Division of Immunology, Department of Pathology, University of Cambridge, Cambridge, UK; 46 John V Farman Intensive Care Unit, Addenbrooke’s Hospital, Cambridge, UK

**Keywords:** Pneumonia, Community-acquired, Hospital-acquired, Ventilator-associated, Intensive care unit (ICU), Antimicrobials, Bronchoscopy, Surveys and questionnaires

## Abstract

**Background:**

Pneumonia remains a significant global health concern, particularly among those requiring admission to the intensive care unit (ICU). Despite the availability of international guidelines, there remains heterogeneity in clinical management. The D-PRISM study aimed to develop a global overview of how pneumonias (i.e., community-acquired (CAP), hospital-acquired (HAP), and Ventilator-associated pneumonia (VAP)) are diagnosed and treated in the ICU and compare differences in clinical practice worldwide.

**Methods:**

The D-PRISM study was a multinational, survey-based investigation to assess the diagnosis and treatment of pneumonia in the ICU. A self-administered online questionnaire was distributed to intensive care clinicians from 72 countries between September to November 2022. The questionnaire included sections on professional profiles, current clinical practice in diagnosing and managing CAP, HAP, and VAP, and the availability of microbiology diagnostic tests. Multivariable analysis using multiple regression analysis was used to assess the relationship between reported antibiotic duration and organisational variables collected in the study.

**Results:**

A total of 1296 valid responses were collected from ICU clinicians, spread between low-and-middle income (LMIC) and high-income countries (HIC), with LMIC respondents comprising 51% of respondents. There is heterogeneity across the diagnostic processes, including clinical assessment, where 30% (389) did not consider radiological evidence essential to diagnose pneumonia, variable collection of microbiological samples, and use and practice in bronchoscopy. Microbiological diagnostics were least frequently available in low and lower-middle-income nation settings. Modal intended antibiotic treatment duration was 5–7 days for all types of pneumonia. Shorter durations of antibiotic treatment were associated with antimicrobial stewardship (AMS) programs, high national income status, and formal intensive care training.

**Conclusions:**

This study highlighted variations in clinical practice and diagnostic capabilities for pneumonia, particularly issues with access to diagnostic tools in LMICs were identified. There is a clear need for improved adherence to existing guidelines and standardized approaches to diagnosing and treating pneumonia in the ICU.

*Trial registration* As a survey of current practice, this study was not registered. It was reviewed and endorsed by the European Society of Intensive Care Medicine.

**Graphical abstract:**

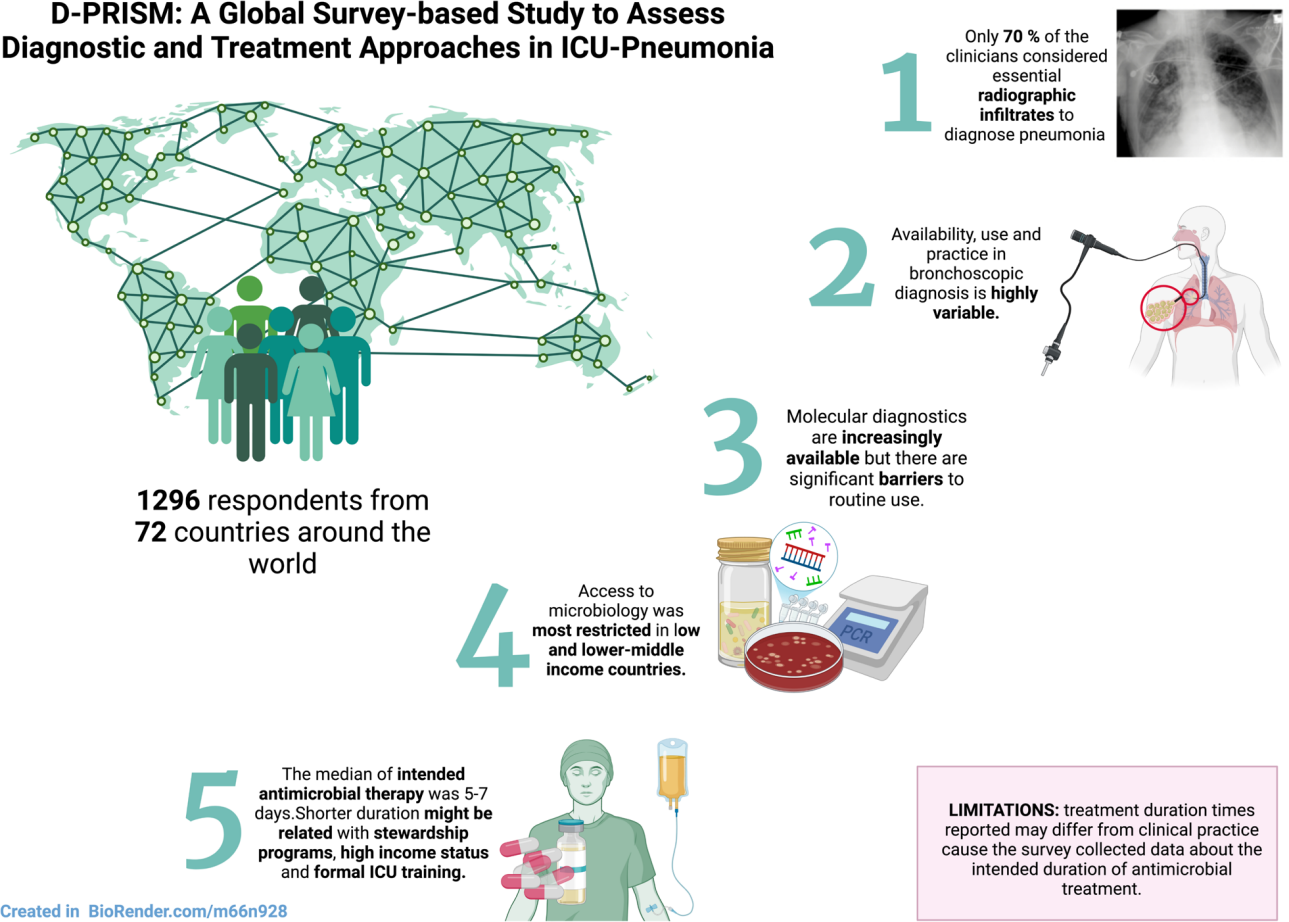

**Supplementary Information:**

The online version contains supplementary material available at 10.1186/s13054-024-05180-y.

## Background

Pneumonia remains a significant global health concern, particularly among critically ill patients requiring intensive care unit (ICU) admission [1–4]. Patients admitted to the ICU present with broad types of lower respiratory tract infections such as community-acquired pneumonia (CAP), hospital-acquired pneumonia (HAP), ventilator-associated tracheitis (VAT), and ventilator-associated pneumonia (VAP); each presentation has distinct challenges in diagnosis and treatment [5–7]. Pneumonia is responsible for a substantial healthcare burden, with estimated costs reaching 10 billion US dollars annually and accounting for more than 2.5 million deaths a year worldwide [1, 6]. Several international guidelines have been published on diagnosing and managing CAP, HAP, and VAP [6, 8–14]; however, the clinical application of these guidelines remains inconsistent due to various factors, including insufficient education, guideline complexity, and organizational barriers contributing to poor adherence among healthcare providers. A lack of training and experience can lead to inconsistent application of guidelines [9, 10, 15, 16].

Although the fundamental definition of pneumonia, namely the presence of alveolar inflammatory infiltration triggered by an infecting organism, has remained constant, the assessment methods have changed over time, and with them, our understanding of clinical disease. The lack of immediate and sensitive pathogen diagnostics leads to broad syndromes that predict likely organisms, with CAP and HAP being the most commonly used clinical diagnoses [11]. Additional classifications have been promulgated, including frailty, healthcare exposure, and immunocompetence [12–14]. The emergence of newer technologies, such as lung ultrasound (LUS), allows sensitive bedside assessment [17–19]. Syndromic molecular tests allow for the rapid evaluation of the presence of pathogens [15, 20–23]. These newer assessment modalities require specific equipment, which comes at the cost of both capital investment and training. Each also brings particular challenges, such as inter-operator variability (for LUS), risk of detection of colonising organisms, and missed detection of off-panel organisms for molecular pathogen detection [24].

Despite the prevalence and clinical significance of pneumonia in the ICU, guidelines on the clinical definition, diagnostic investigation, and management show divergence across the globe [25]. There is also a shortage of evidence regarding comparative clinical practice across the domains of diagnosis and management. Identifying the causative microbes in CAP, HAP, and VAP in the ICU remains challenging, with most cases having no identified pathogen [21, 26–30]. The scarcity of evidence regarding practice is most pronounced in low and middle-income countries where the incidence and mortality of pneumonia is greatest [1] and where the resource limitations may further impact diagnostic accuracy and treatment strategies [31, 32].

The D-PRISM study was conducted as a multinational effort to address these critical gaps in the existing literature. It aimed to provide a comprehensive assessment of the clinical practices employed by critical care clinicians in diagnosing and treating pneumonia admitted to the ICU, with a particular focus on CAP, HAP, and VAP. Due to uncertainties around the definition and diagnosis of VAT, we did not include this in our survey, which was intended to focus specifically on pneumonia [33]. The study sought to evaluate the applicability and adherence to existing clinical guidelines, assess the challenges encountered in clinical and microbiological diagnosis, and explore variations in clinical practices by illuminating these crucial aspects.

## Methods

This multinational cross-sectional study conducted an online self-administered questionnaire (SurveyMonkey, Momentive, San Mateo, CA) to intensive care clinicians globally between September and November 2022, with responses from 72 countries. The European Society of Intensive Care Medicine (ESICM) invited all its members to participate in the study, and distribution was further achieved through national coordinators identified by the steering committee. This survey did not collect any data that would allow for the identification of respondents. Participation in the survey was entirely voluntary, and respondents provided consent for using their anonymized answers by choosing to participate. IP address registration was used to prevent multiple participation. The IP addresses were removed prior to analysis and not used for any other purpose, ensuring full compliance with data protection and privacy regulations. As an anonymized survey of clinical practice without individual patient data, the UK Health Research Authority waived the requirement for research ethics approval and formal written consent.

### Questionnaire

The D-PRISM questionnaire was created by a steering committee of ESICM members with clinical and research experience in diagnosing and managing severe pneumonia. The survey was translated and available in 10 languages, including Arabic, German, Greek, English, Spanish, French, Portuguese, Russian, Turkish, and Chinese. All the questions were discussed and selected by group consensus and contributed to the current state of practice in severe pneumonia (i.e., pneumonia managed in the ICU), with the final survey being piloted within the steering committee. To ensure accuracy and clarity, national coordinators identified by the steering committee reviewed the translations. The final questionnaire had 40 questions and was divided into five sections that evaluate the professional profile (11 questions), diagnosis and treatment of CAP (5 questions), diagnosis and treatment of HAP (6 questions), diagnosis and treatment of VAP (6 questions), and availability of microbiology diagnostic tests (12 questions). It included open, closed, multiple choice, and Likert scale questions. Where frequency of assessment (‘always’, ‘mostly’, ‘sometimes’, ‘never’) was examined, ‘routine use’ was considered to be ‘always’ or ‘mostly’. This questionnaire was not externally validated as its purpose was to address and collect the current state of medical practice in managing severe pneumonia in the ICU. The complete questionnaire is available in the supplementary material.

### Pneumonia definition

All the definitions were provided to the participants before the survey competitions and are based on the current American Thoracic Society—Infectious Diseases Society of America (ATS/IDSA) clinical guidance [5, 6, 15], similar to the definitions used in other national and multinational guidelines.

*Community-acquired pneumonia (CAP)* was defined as pneumonia or suspected pneumonia present at hospital admission or manifesting within 48 h of hospital admission.

*Hospital-acquired pneumonia (HAP)* was defined as pneumonia or suspected pneumonia that does not present at hospital admission and develops at least 48 h after hospital admission, and it includes non-ventilated ICU patients.

*Ventilator-associated pneumonia (VAP)* was defined as pneumonia or suspected pneumonia that does not present at ICU admission and develops at least 48 h after initiation of mechanical ventilation. It included patients who developed symptoms within 48 h of extubation.

### Participants

We used a convenience sampling strategy; the participant responders were physicians and physicians in training who work in ICUs and were willing to participate in the study. The study’s aim, scope, and confidentiality were explained to them. All the answers were anonymous and collected in an Excel worksheet. No remuneration or incentive was offered to participate. We cannot estimate a denominator as staffing figures do not exist for all nations surveyed.

### Statistical analysis

All the categorical variables were presented in relative and absolute frequencies. The continuous variables were presented as medians and interquartile ranges (IQR) or mean and standard deviation (S.D.) based on their normality evaluated with the Kolmogorov–Smirnov test (*p* < 0.05). The results were analyzed and described by the type of pneumonia and the World Bank country classification. The questionnaires with more than 25% unanswered questions were excluded from the analysis. All other data fields were analyzed as recorded; no imputation was undertaken for missing data. We performed a multifaceted approach encompassing both bivariable and multivariable analyses to assess factors associated with the intended duration of antibiotic treatment. Variables identified in the univariable analysis with a *p* value < 0.20 were included in the multivariable model [34]. We carried out multivariable analyses, utilizing multiple regression models to assess the impact of multiple independent variables on a dependent variable. Microsoft Excel and SPSS version 29.0 statistical package performed all the descriptive analyses.

We followed the guidelines outlined in the Consensus-Based Checklist for Reporting of Survey Studies (CROSS) [35], with the CROSS checklist in the supplemental section.

## Results

**Fig. 1:**
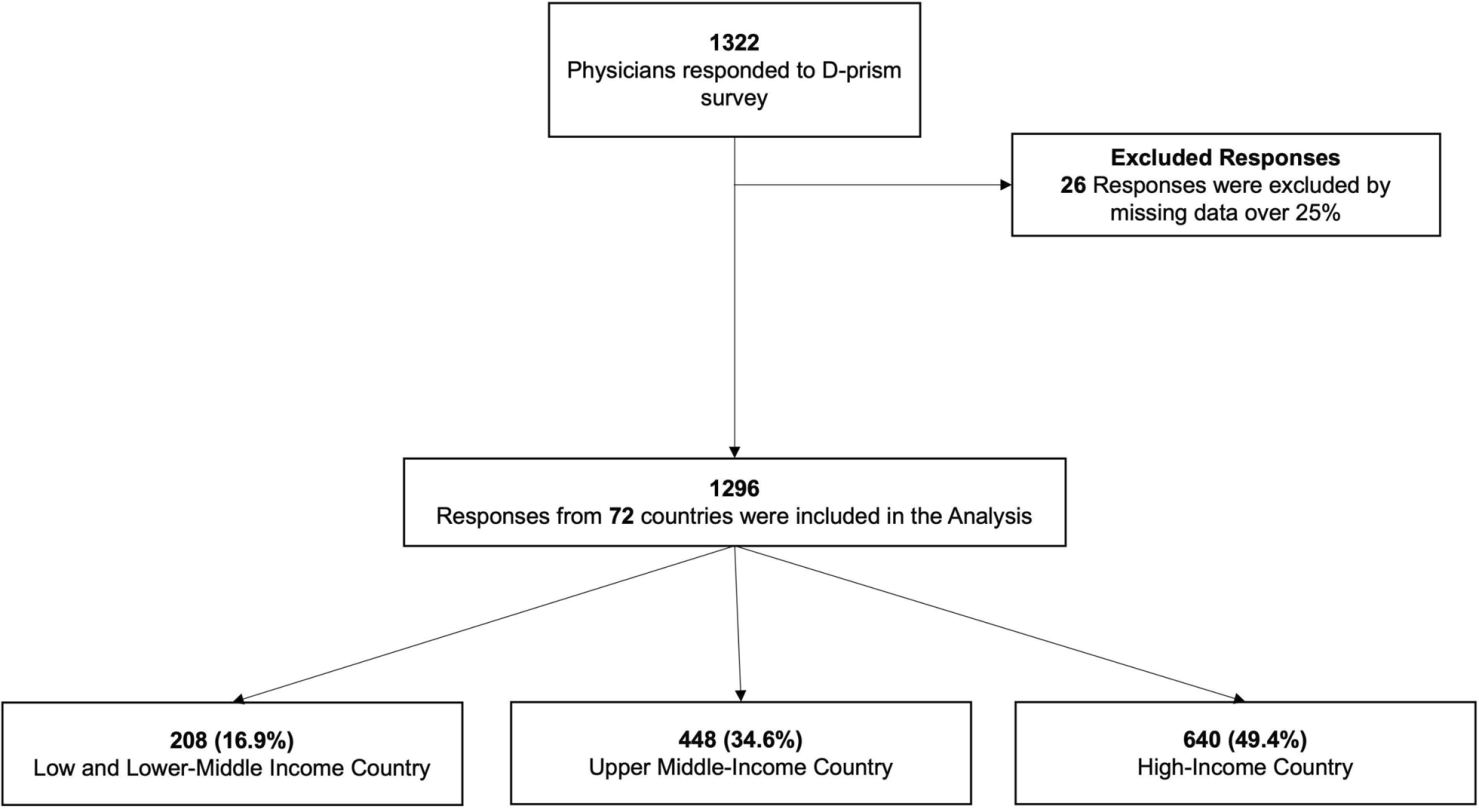
Study flow chart

In total, 1322 responses were collected from 72 countries, 26 invalid responses were excluded 25% or greater missing data (Fig. 1). Just under half of the respondents were from high-income countries, 35% were from upper-middle-income countries, and 16% were from low- and lower-middle-income countries (Table 1, Figs. S1, S2). Notable features are the dominance of respondents from teaching or university hospitals (71%) and mixed medical/surgical units (78%), with this dominance noted across all national income levels (Table S1). The criteria discriminated by World Bank income classification are shown in Table S2. Hospitals were generally mid-sized, with a median of 526 beds and 20 ICU beds. Specialization in Intensive Care Medicine was reported by 79%, with just over 1/3rd of these reporting an additional specialization. Respondents were mainly experienced, with 70% having five or more years of post-graduate ICU experience (Table 1). Having an antimicrobial stewardship (AMS) program and/or pneumonia-specific antibiotic protocols and/or local guidelines were widely reported, being present in 80% and 79% of hospitals, respectively (Table 1).

**Table 1:** Characteristics of the respondents

Characteristics	N = 1296
*Region*	
Asia	484 (37%)
Europe	406 (31%)
Central and South America	154 (12%)
Sub-Saharan Africa	86 (7%)
Australasia	58 (5%)
North America	66 (5%)
North Africa	42 (3%)
*Income level*	
High-income	640 (49%)
Upper-middle income	448 (35%)
Lower-middle income	201 (16%)
Low-income	7 (0.5%)
*Hospital*	
University/teaching hospital	913 (71%)
Community district hospital	338 (26%)
Remote and rural hospital	41 (3%)
*ICU-unit*	
General ICU (medical and surgical)	1004 (78%)
Medical ICU	161 (12%)
Surgical ICU	73 (6%)
Cardiac ICU	34 (3%)
Neuro ICU	24 (2%)
*Hospital size*	
Hospital Beds, median (IQR)	526 (264–1000)
ICU beds, median (IQR)	20 (12–31)
*Protocol of hospital*	
Antimicrobial stewardship program	1023 (79%)
Local Antimicrobial Guidelines	1033 (80%)
*Medical specialist*	
Intensive care physician	1005 (78%)
Anaesthesiologist	107 (8%)
Respiratory Physician	79 (6%)
Internal medicine physician	34 (3%)
Infections disease physician	25 (2%)
Emergency physician	8 (0.6%)
Others*	3838 (3%)
*Postgraduate ICU experience*	
> 10 years	610 (47%)
5–10 years	303 (23%)
< 5 years	381 (29%)

*Other medical specialities, including cardiology (3), family physician (4), gastroenterologists (1), geriatricians (1), haematologists (1), nephrologists (3), surgeons (9), Orthopaedics (1), Rehabilitation physician (1), Physician not otherwise specified (10) paediatricians (3), medical student (1)

### Community-acquired pneumonia

**Fig. 2:**
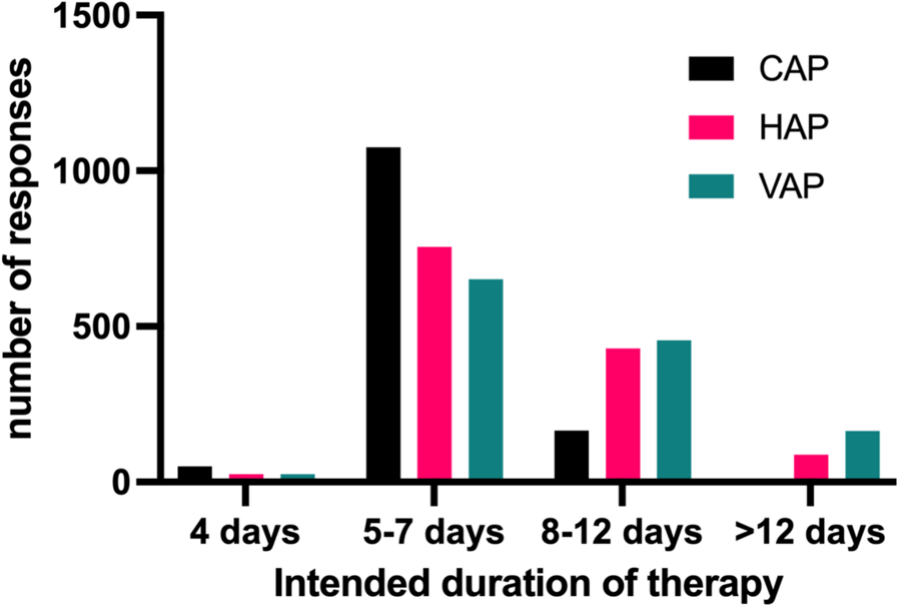
Duration of antibiotic regimen by disease. *CAP* Community-acquired pneumonia, *HAP* Hospital-acquired pneumonia, *VAP* Ventilator-associated pneumonia

Although respondents almost universally assessed clinical and radiological features in patients with suspected CAP (Table 2), only 65% considered the presence of positive findings in both essential for the diagnosis (individually, 64% reported clinical criteria essential, 71% radiological criteria). The use of lung ultrasound (LUS) was reported by 29%, with a similar proportion reporting a combination of clinical presentation and LUS for CAP diagnosis (Table 2). Regarding the samples taken at diagnosis, sputum or endotracheal aspirate was the most common microbiological sample taken by 83% of respondents. In mechanically ventilated patients, blind mini-bronchoalveolar lavage (mini-BAL) was reportedly used by 33%. Bronchoalveolar lavage (BAL) was routinely used in 29% of mechanically ventilated patients and 11% of non-mechanically ventilated patients (Table 2). Blood culture was very commonly (86%) reported as “always” sampled (Table 2). Regarding antimicrobial treatment, 64% of respondents’ initial empiric regimens included dual therapy with a macrolide, 28% used monotherapy, and 8% (105/1296) used dual therapy, including a non-macrolide (Table 3). If the patient responded to the initial regimen, the reported intended duration of treatment was 5 to 7 days in 82% (Fig. 2, Table 4).

**Table 2:** Respondents’ assessment rates for diagnostic criteria

Diagnostic criteria	CAP n = 1296	HAP n = 1296	VAP n = 1296
Clinical presentation**	1235 (95%)	1234 (95%)	1218 (94%)
*Radiological criteria*			
Chest X-ray or CT scan**	1257 (97%)	1259 (97%)	1244 (96%)
Lung ultrasound**	379 (29%)	405 (31%)	443 (34%)
*Clinical and radiological criteria*			
Clinical presentation and Chest X-ray or CT scan*	845 (65%)	747 (58%)	742,742 (57%)
Clinical presentation and lung ultrasound*	362 (28%)	115 (9%)	151,151 (12%)
*Laboratory tests performed at diagnosis*			
Sputum or endotracheal aspirate**	1070 (83%)	1153 (89%)	1174 (91%)
Blind mini-bronchoalveolar lavagelavage**	430 (33%)	507 (39%)	527 (41%)
Bronchoalveolar lavage**	381 (29%)	500,500 (39%)	521,521 (40%)
Blood culture*	803 (62%)	835,835 (64%)	847,847 (65%)

Clinical presentation included the following signs and symptoms: positive findings on auscultation such as bronchi, crepitations, wheeze, breathlessness, purulent sputum, impaired oxygenation or ventilation; Radiological findings included: CXR or C.T. showing lobar infiltration or bronchogram or diffuse or patchy shadowing; Sonological findings include USS showing consolidation or bronchogram

*Frequencies reported only include the “always” response

**Frequencies reported the combination of “always” and “mostly” responses

**Table 3:** Antimicrobial regimen used in CAP patients

Empirical treatment CAP n = 1296	
Antibiotic regimen	
Monotherapy	357 (27.5%)
Dual therapy with macrolide	827 (63.8%)
Dual therapy with non-macrolide	105 (8.1%)
Not answered	7 (0.5%)

**Table 4:** Intended treatment duration

Intended antimicrobial duration	All cohort	High income countries	Upper-middle-income countries	Low and low-middle income countries
*CAP*				
< 5 days	51 (3.9%)	16 (2.5%)	20 (4.5%)	15 (7.2%)
5–7 days	1059 (81.7%)	546 (85.3%)	355 (79.2%)	158 (75.9%)
> 7 days	174 (11.1%)	71 (11.0%)	71 (15.8%)	32 (15.4%)
Not answer	12 (0.9%)	7 (1%)	2 (0.5%)	3 (1.5%)
*HAP*				
< 5 days	25 (1.9%)	6 (0.9%)	8 (1.8%)	11 (5.2%)
5–7 days	743 (57.3%)	421 (65.8%)	226 (50.4%)	96 (46.1%)
> 7 days	515 (39.7%)	207 (32.3%)	211 (47.1%)	97 (46.6%)
Not answer	13 (1.0%)	6 (1%)	3 (0.6%)	4 (2%)
*VAP*				
< 5 days	24 (1.8%)	5 (1%)	10 (2.2%)	9 (4.3%)
5–7 days	643 (49.6%)	380 (59.3%)	186 (41.5%)	77 (37.1%)
> 7 days	615 (47.4%)	250 (39.1%)	246 (54.9%)	119 (57.2%)
Not answer	14 (1.0%)	5 (1%)	6 (1.2%)	3 (1.5%)

*CAP* Community-acquired pneumonia, *HAP* Hospital-acquired pneumonia, *VAP* Ventilator-associated pneumonia

### Hospital-acquired and ventilator-associated pneumonia

Regarding HAP and VAP, the frequency of assessments was similar to those reported for CAP. However, the respondents reported a lower frequency of a combination of clinical presentation and radiological signs being used to confirm diagnosis (58% for HAP and 57% for VAP). Usage of clinical presentation and LUS were also lower in these two conditions (9% for HAP and 12% for VAP, respectively) (Table 2). Sputum or endotracheal aspirates were the most requested microbiological sample, reaching 89% for HAP and 91% for VAP. More invasive sampling in the form of blind mini-BAL or formal broncho-alveolar lavage approached 40% for both HAP and VAP. In contrast to CAP, 36% of the respondents considered the usage of blood cultures for HAP and VAP unnecessary (Table 2). Regarding empiric antimicrobial therapy, 39% of respondents preferred dual treatment with coverage for resistant organisms in HAP, whilst for VAP, the rate increased to 48%. The remaining clinicians indicated selective use of dual therapy in patients at higher risk of multi-drug resistant organisms (Table 5). Over half of all respondents reported antibiotic ‘time-outs’ or mandated reviews at 48–72 h (Table S3). Relative to CAP, where 87% reported the duration of treatment as 5–7 days, the duration of antimicrobial therapy following an initial improvement was longer in HAP and VAP, with 40% and 47%, respectively, reporting a duration of greater than 7 days (Fig. 2, Table S2).

**Table 5:** The antimicrobial regimen used in HAP and VAP patients

Antibiotic regimen	HAP n = 1296	VAP = 1296
Monotherapy for all patients	209 (16.1%)	163 (12.5%)
Monotherapy for low risk of resistant organisms	572 (44.1%)	511 (39.4%)
Dual therapy, including coverage for resistant organisms (e.g. MRSA/MDR pseudomonas) for all patients	510 (39.3%)	616 (47.5%)
N/A	5 (0.3%)	6 (0.4%)

### Factors associated with the treatment duration

Univariable analysis was performed to identify factors associated with the intended treatment duration. The multivariable model included variables with a *p* < 0.20 in the initial univariable analysis [34]. Odds ratios (OR) were calculated based on the exponentials of the coefficients obtained by the final model and presented in forest plots (Fig. S3 panels A–C). CAP, HAP, and VAP responses were compared with an intended treatment duration of less than or equal to 7 days. The models demonstrated a good fit as indicated by a Hosmer and Lemeshow test *p* value of (*p* = 0.76) for CAP, (0.08) for HAP, and (*p* = 0.42) for VAP. Also, an acceptable discriminatory power as indicated by an AUC ROC value of (0.65) for CAP, (0.67) for HAP, and (0.68) for VAP. For CAP, the characteristics associated with treatment ≤ 7 days (Odds Ratio [95% Confidence Interval] *p* value) were being an intensivist (1.47 [1.00–2.15] *p* = 0.04), having an AMS program (1.93 [1.33–2.78] *p* < 0.001), diagnostic criteria that combined clinical presentation and radiological requirements (1.43 [1.02–2.00] *p* = 0.034) and dual therapy with a macrolide (1.78 [1.28–2.47] *p* < 0.001) (Fig. S3A, Table S4). For HAP, the factors associated with treatment less than 7 days (Odds Ratio [95% Confidence Interval] *p* value) were being an intensivist (1.46 [1.09–1.96] *p* = 0.01), having an AMS program (1.79 [1.35–2.39] *p* = < 0.001), being from a high-income country (1.45 [1.13–1.87] *p* = 0.004), having an antibiotic time out (1.46 [1.15–1.85] *p* = 0.002) and the use of monotherapy (2.55 [1.72–3.77] *p* < 0.001). We also identified that for HAP standard dual-therapy covering methicillin-resistant *Staphylococcus aureus* (MRSA), multi-drug resistant pathogens (MDRP) and *Pseudomonas* spp. (0.68 [0.53–0.87] *p* = 0.003) was a factor related to a treatment duration of more than 7 days (Fig. S3B, Table S5). For VAP the factors associated with treatment less than 7 days were having an AMS program (1.74 [1.30–2.33] *p* < 0.001), being from high-income country (1.62 [1.26–2.07] *p* < 0.001), having an antibiotic time-out (1.46 [1.16–1.85] *p* = 0.001) and the use of monotherapy (3.22 [2.09–4.97] *p* < 0.001) (Fig. S3C, Table S6).

### Bronchoscopy

In low and lower-middle-income countries, 61% of respondents reported having bronchoscopy available, but only 29% could perform this exam at any time (24-h availability), with 20% reporting restricted daily availability (Table 6). By contrast, bronchoscopy availability was nearly universal in high-income nations (97%), with 76% having 24-h availability. Upper-middle-income nation respondents indicated similar overall availability of bronchoscopy at 90%, but more restricted hours and days than those from high-income nations (Table 6).

**Table 6:** Availability of diagnostic tests in countries classified by The World Bank income classification

Diagnostic test	World Bank classification		
	High-income n = 640 (%)	Upper-middle income n = 448 (%)	Low and lower-middle income n = 208 (%)
Bronchoscopy	621 (97%)	406 (91%)	126 (61%)
*Availability of bronchoscopy*			
Always	488 (76%)	230 (51%)	56 (26%)
Specific hours every day	86 (13%)	84 (19%)	36 (17%)
Specific weekdays	47 (7%)	92 (20%)	33 (16%)
*Who performs the bronchoscopy*			
Intensive care specialist	547 (86%)	190 (42%)	66 (31%)
Respiratory Physician from outside of the ICU unit	66 (10%)	136 (30%)	54 (25%)
Not answered	8 (1%)	80 (18%)	6 (3%)
*BAL*			
Level of training performing			
Formal training	233 (36%)	188 (42%)	54 (26%)
Informal training	318 (50%)	125 (28%)	69 (33%)
No training	67 (11%)	108 (24%)	53 (26%)
*Microbiological test*			
Not available			
Pneumococcal urinary test	135 (21%)	293 (65%)	150 (72%)
Legionella urinary test	40 (6%)	224 (50%)	127 (61%)
Single organism PCR	231 (36%)	173 (39%)	94 (45%)
Multiplex PCR test	95 (15%)	100 (22%)	79 (38%)
Available			
Pneumococcal urinary test	480 (75%)	83 (19%)	27 (13%)
Legionella urinary test	586 (92%)	162 (36%)	47 (23%)
Single organism PCR	318 (50%)	187 (42%)	74 (36%)
Multiplex PCR test	501 (78%)	285 (64%)	91 (44%)
*Conventional cultures*			
Not available			
Sputum	3 (0.4%)	7 (2%)	9 (4%)
Blind mini BAL	88 (14%)	60 (13%)	25 (12%)
BAL	1 (0.1%)	13 (3%)	24 (12%)
Blood	0 (0%)	0 (0%)	9 (4%)

Training in bronchoscopy was variable: among high and upper-middle-income country respondents, 36% and 43% reported formal and informal training, respectively (Table 6), with similar rates between fully qualified specialists and trainees/residents (Table 7). Consistent with trends in the availability of bronchoscopy, those in low/lower-middle-income nations were less likely to report formal or informal training (Table 6).

**Table 7:** Training in bronchoscopy description

Type of training	Degree seniority			BAL characteristics	
	Consultant/specialist in intensive care n = 1048	Trainee/resident in intensive care n = 202	Other n = 46	Confidence in performing BAL, median (IQR)*	Instilled volume (mL) used for BAL, median (IQR)**
Formal training in BAL	392 (37%)	68 (34%)	15 (33%)	8 (7–10)	50 (20–100)
Informal training in BAL	433 (41%)	65 (32%)	14 (30%)	7 (5–8)	30 (15–60)
No training in BAL	154 (15%)	60 (30%)	14 (30%)	2 (0–5)	20 (10–50)
Training in BAL not reported	69 (7%)	9 (4%)	3 (7%)	4 (0–8)	20 (10–100)

*BAL* Bronchoalveolar lavage

*Confidence in performing BAL was measured on a self-perception scale from (0 to 10), with 0 being no confidence at all and 10 being the highest confidence value

**The standard BAL approach involves instilling the sterile saline in increments of 20–50 mL per aliquot, usually in three to five separate aliquots, to achieve adequate lavage and sampling

Where bronchoscopy was available, 58% of respondents reported intensivists as the sole providers, with upper and low/low-middle-income respondents more commonly relying on specialists from outside the ICU for service provision (Table 6). Confidence in bronchoscopy was assessed by a 10-point Likert scale, which demonstrated a relationship with the degree of training, as did specialisation in respiratory medicine (Table 7). The lavage volume also varied with self-reported training status and specialisation in respiratory medicine (Table 7). A weak but significant positive correlation existed between confidence in bronchoalveolar lavage and volume used (r = 0.25 *p* < 0.001). Notably, even amongst the most confident and highly trained bronchoscopists, the median lavage volume was 50 ml, well below the 100–200 ml recommended in bronchoscopy guidelines to achieve adequate alveolar lavage [36].

### Microbiological tests

Conventional microbiological cultures are reported quantitively for sputum by 54% of responding institutions, for blind mini-BAL by 43%, for BAL by 57%, and for blood by 48%. While sputum and blood cultures were almost universally available (98% and 99% overall), there was a noticeable income-related disparity, with these tests being less accessible in low and lower-middle-income ICUs (Tables 6 and S7). This gap was even more pronounced for deep lung sampling techniques, where 13% of respondents reported mini-BAL being unavailable, and 3% indicated BAL was unavailable (Tables 6 and S7).

The use of molecular microbiological tests varied between countries. In low-middle-income and upper-middle-income countries, the availability of multiplex molecular tests was relatively frequently reported at 44% and 64%, respectively. The Legionella urinary test was the most reported in high-income countries at 92% (Table 6). The pneumococcal urinary antigen test was available by 75%; however, low/lower-middle-income countries reported low availability of these assays—only 22% had access to the Legionella antigen test and 13% to the Pneumococcal antigen test. Regarding barriers to using multiplex testing, 44% of respondents reported a barrier to access of some type, of which the most common was the cost of the test, reported by 69%. The named multiplex test most frequently reported was the BioFire film array (reported by 23%), although 31% did not know which specific test was used at their institution (Fig. S4).

## Discussion

The D-PRISM study provides insights into the current clinical diagnosis and management of pneumonia in the ICU. To the best of our knowledge, it is the largest and most geographically widespread study of clinical approaches to severe pneumonia yet reported. We found similar approaches to clinical and radiological diagnosis by national income level but with more divergence regarding bronchoscopic sampling and microbiological testing. Antimicrobial duration was associated with several unit-based factors.

There are significant variations in clinical practices among critical care clinicians. While assessing clinical and radiological features was nearly universal, 1/3rd of clinicians did not feel that the radiographic feature of pulmonary infiltrates was essential for diagnosis, whether for HAP, VAP, or CAP. This reflects results from a single-nation VAP-only study where 33% of respondents did not require radiographic findings to diagnose pneumonia [37]. It should be noted that while most guidelines advocate radiographic evidence of infiltration for diagnosing CAP [38, 39], HAP, and VAP [5, 6, 40, 41], several HAP and VAP guidelines identify issues with the sensitivity and specificity of radiographic evaluation [5, 6, 41] and advise starting antibiotics based on clinical suspicion alone. This may reflect clinicians’ lack of certainty in using imaging as a confirmatory tool. Notably, however, most of the randomized clinical studies carried out to treat VAP and CAP include the radiological criteria for diagnosis as essential and do not encourage the start of empirical treatment without confirming radiographic infiltrates [42, 43]. Of note, in our study, only 60% of respondents reported adhering to the recommended diagnostic criteria, highlighting a potential gap between guideline recommendations and clinical practice, which could drive increased use of antibiotics. The absence of radiographic assessment will also make the differentiation of VAT from pneumonia more challenging. The role of antibiotic prescribing in VAT remains uncertain and controversial [33].

The frequency of microbiological sampling differed between the sub-types of pneumonia, with 52% of respondents always taking samples in CAP but rising to 62% and 74% in HAP and VAP, respectively. However, this was well short of the 100% microbiological sampling in the guidelines for severe CAP [8, 38, 39] and HAP/VAP [5, 6, 40, 41]. Microbiological testing is recommended to guide antimicrobial therapy for severe CAP and nosocomial lower respiratory infections, such as HAP and VAP. However, the strength of these recommendations is generally conditional with low quality of evidence, reflecting the balance between potential benefits and the limitations of current diagnostic methods [15, 44]. It should also be noted that only in 38% of cases of pneumonia is it possible to identify the microorganism causing pneumonia [26] and that truly unbiased molecular detection remains a research tool [45]. The higher sampling rates in HAP and VAP cases may reflect increasing diagnostic uncertainty where non-infectious mimics are more common [46]. The use of deeper lung samples, including mini-BAL and bronchoscopic BAL, was also more common in ventilated HAP and VAP than ventilated CAP, perhaps reflecting greater concern regarding contamination of the proximal respiratory tract in those who have been hospitalized or ventilated for longer [47, 48]. Notably, however, guideline advocacy for invasive diagnostic techniques in this area is caveated by uncertainty as to the balance of risks of bronchoscopy against the benefits of improved antimicrobial stewardship [5, 41]; indeed, the U.S. guidelines weakly advise against invasive sampling [6].

Conventional microbiology was widely used, although it is notable that a significant proportion of low-and-middle-income respondents did not have this diagnostic modality available. The influence of the recent COVID-19 pandemic could be detected through the widespread availability of single-plex polymerase chain reaction testing. Although multiplex testing was also more widely available than anticipated, this may reflect the respondents' teaching/university hospital bias. While multiplex PCR offers better pathogen detection and more precise treatment options, its impact on patient outcomes remains underexplored. Research suggests that multiplex PCR testing can help start the correct antibiotics earlier and reduce the need for broad-spectrum treatments. However, the strength of this evidence varies; not all studies show the same level of benefit, and it is not yet clear whether this translates into better clinical or microbiological outcomes [23, 49, 50]. Furthermore, although we did not ascertain how frequently these tests were used, many respondents reported barriers to use, especially in lower-income settings where most respondents reported cost and reagent availability as barriers.

Although bronchoscopic lavage was reported as a routine ('always or mostly') diagnostic test in 40% of HAPs and VAPs, training in this technique is highly variable, and a lack of formal training was associated with lower operator confidence and the use of smaller lavage volumes. For lavage to sample the alveolar space (primary site of pneumonia), a sufficient volume is required to form a continuous column from the scope to the alveoli, typically estimated to be at least 100 ml in an adult [36]. The median volume used was 30 ml, and 77% of respondents used < 100 ml, which suggests that in most patients, the alveolar space is not being adequately sampled, and as such, results may not be comparable to clinical studies where high-volume lavage is used [47, 51].

The duration of intended antimicrobial therapy was reported to be 5–7 days for CAP, in line with recommendations from the ATS/IDSA guidelines [15], with longer durations reported for the treatment of HAP and VAP, likely reflecting concerns regarding resistant organisms, especially non-fermenting Gram-negatives such as Pseudomonas aeruginosa [52]. However, it is possible that real-world practice differs from intended antimicrobial duration. A recent study by Yi et al. found that the median duration of antimicrobial therapy was 9.5 days, with 70% of cases being assessed as excessive duration [53]. Antimicrobial stewardship interventions have previously demonstrated a reduction in the duration of therapy and hospital length of stay, improving antimicrobial-guided treatment and adherence to local and international guidelines [54–58]. Respondents from centres that reported the presence of an AMS program, which is a structured programme of collection, analysis, and feedback of data around antimicrobial prescribing and antibiotic stewardship interventions, tended to have shorter intended durations of antimicrobial courses*.* This effect was also seen with antibiotic time-out at 48–72 h, a structured intervention to formally review the requirement for ongoing antibiotic therapy after the time-period indicated. However, the relationships identified by regression analysis need to be considered exploratory given the convenience sample nature of this study. Although the higher rates of bacterial resistance in lower-middle and low-income countries might be expected to impact antimicrobial therapy duration, in our study, the length of intended therapy for CAP did not differ by national income status. However, higher national income status was associated with shorter durations for HAP and VAP. This data cannot determine whether this apparently increased duration was driven by perceived or actual rates of antimicrobial resistance. In line with recommendations from ATS/IDSA [38] and the British Thoracic Society guidelines [59], most respondents reported using dual therapy with a macrolide as the first line for CAP. Perhaps reflecting the uncertainty and reported risk of harm from routine use of dual therapy in HAP and VAP [60], treatment here was more cautious, with 40% reporting dual therapy for all patients and 44% reporting selective use when patients were deemed at high risk of multi-drug resistant organisms.

This study has several advantages over previous studies in this area, as the number of respondents and coverage from 72 countries increased the representativeness of the findings. However, it has some limitations, notably that the response rate cannot be calculated as it was distributed beyond members of the ESICM. Furthermore, the responses are skewed towards academic centres and may not reflect practice in other settings. Although we took measures to ensure linguistic accuracy and cultural relevance, the absence of a formal validation process for the survey may introduce variability in the interpretation of questions across different languages. Additionally, only 16% of the respondents were from low and lower-middle-income countries. This disparity might reflect unequal access to internet-based resources and lower participation in international societies. The study did not collect data about steroid use or specific local antimicrobial regimens. Furthermore, we restricted our analysis to the ‘conventional pharmacopoeia’ and did not explore the use of herbal or other traditional remedies. Likewise, the role of other specialists as consultants from outside the ICU was not evaluated. We did not break pneumonia down into illnesses arising from different classes of organisms, e.g., viral, atypical, and extracellular bacteria, as this data is frequently unavailable at the time of initial diagnosis. However, it is possible that initial diagnostic and clinical management may differ depending on the suspected or confirmed aetiological agent. The study was based on clinicians’ self-assessment of practice and, therefore, cannot be certain of the reports’ reliability. Treatment duration times reported may differ from clinical practice as the survey only collected data regarding the intended duration of antimicrobial treatment. Despite these limitations, this study addresses various issues that provide valuable insights into clinicians' behaviour and decision-making processes. These findings invite further exploration of adherence to, and the applicability of, international guidelines through studies involving patient-level data.

## Conclusions

In conclusion, we found widespread variations in practice regarding diagnosing severe pneumonia in critical care. While clinicians tend to use similar clinical and radiological criteria for both CAP and HAP/VAP, they show more variability in microbiological sampling methods, and many clinicians deviate from the international guidance on diagnostic approaches. There is increasing availability of techniques such as ultrasound and multiplex molecular testing, but their use is far from universal, and considerable barriers remain to routine implementation. There are significant issues with the availability of bronchoscopy in the ICU, with gaps in the training and experience in its use and evidence of suboptimal sampling techniques. The potential to improve standardisation in the diagnosis and management of pneumonia is considerable and presents opportunities in the future to examine the effects of such a standardisation on patient outcomes. This underscores the need for further observational or audit-based research to assess how closely clinical practice aligns with established guidelines more accurately. This could provide a more robust understanding of the reasons behind these variations and help identify potential barriers to guideline implementation.

## Supplementary Information

Additional file 1.

Additional file 2.

Additional file 3.

Additional file 4.

## Data Availability

The dataset supporting the conclusions of this article is included within the article (and its additional files).

## Abbreviations

ICU: Intensive care unit
CAP: Community-acquired pneumonia
HAP: Hospital-acquired pneumonia
VAP: Ventilator-associated pneumonia
LMIC: Low middle-income countries
HIC: High-income countries
LUS: Lung ultrasound
ESICM: The European Society of Intensive Care Medicine
IQR: Interquartile ranges
BAL: Bronchoalveolar lavage
mini-BAL: Mini-bronchoalveolar lavage
AMS: Antimicrobial stewardship
MRSA: Methicillin-resistant Staphylococcus aureus
MDRP: Multi-drug resistant pathogens

## References

[1] Collaborators GBDLRI. Estimates of the global, regional, and national morbidity, mortality, and aetiologies of lower respiratory infections in 195 countries, 1990–2016: a systematic analysis for the Global Burden of Disease Study 2016. Lancet Infect Dis. 2018;18(11):1191–210. 30243584 10.1016/S1473-3099(18)30310-4 PMC6202443

[2] Zaragoza R, Vidal-Cortes P, Aguilar G, Borges M, Diaz E, Ferrer R, et al. Update of the treatment of nosocomial pneumonia in the ICU. Crit Care. 2020;24(1):383. 32600375 10.1186/s13054-020-03091-2 PMC7322703

[3] Spalding MC, Cripps MW, Minshall CT. Ventilator-associated pneumonia: new definitions. Crit Care Clin. 2017;33(2):277–92. 28284295 10.1016/j.ccc.2016.12.009 PMC7127414

[4] Aliberti S, Reyes LF, Faverio P, Sotgiu G, Dore S, Rodriguez AH, et al. Global initiative for meticillin-resistant *Staphylococcus aureus* pneumonia (GLIMP): an international, observational cohort study. Lancet Infect Dis. 2016;16(12):1364–76. 27593581 10.1016/S1473-3099(16)30267-5

[5] Torres A, Niederman MS, Chastre J, Ewig S, Fernandez-Vandellos P, Hanberger H, et al. International ERS/ESICM/ESCMID/ALAT guidelines for the management of hospital-acquired pneumonia and ventilator-associated pneumonia: Guidelines for the management of hospital-acquired pneumonia (HAP)/ventilator-associated pneumonia (VAP) of the European Respiratory Society (ERS), European Society of Intensive Care Medicine (ESICM), European Society of Clinical Microbiology and Infectious Diseases (ESCMID) and Asociacion Latinoamericana del Torax (ALAT). Eur Respir J. 2017;50(3). 10.1183/13993003.00582-2017 28890434

[6] Kalil AC, Metersky ML, Klompas M, Muscedere J, Sweeney DA, Palmer LB, et al. Management of adults with hospital-acquired and ventilator-associated pneumonia: 2016 clinical practice guidelines by the Infectious Diseases Society of America and the American Thoracic Society. Clin Infect Dis. 2016;63(5):e61–111. 27418577 10.1093/cid/ciw353 PMC4981759

[7] Martin-Loeches I, Reyes LF, Nseir S, Ranzani O, Povoa P, Diaz E, et al. European network for ICU-related respiratory infections (ENIRRIs): a multinational, prospective, cohort study of nosocomial LRTI. Intensive Care Med. 2023;49(10):1212–22. 37812242 10.1007/s00134-023-07210-9 PMC10562498

[8] Martin-Loeches I, Torres A, Nagavci B, Aliberti S, Antonelli M, Bassetti M, et al. ERS/ESICM/ESCMID/ALAT guidelines for the management of severe community-acquired pneumonia. Intensive Care Med. 2023;49(6):615–32. 37012484 10.1007/s00134-023-07033-8 PMC10069946

[9] Salluh JIF, Póvoa P, Beane A, Kalil A, Sendagire C, Sweeney DA, Pilcher D, Polverino E, Tacconelli E, Estenssoro E, Frat JP, Ramirez J, Reyes LF, Roca O, Nseir S, Nobre V, Lisboa T, Martin-Loeches I. Challenges for a broad international implementation of the current severe community-acquired pneumonia guidelines. Intensive Care Med. 2024;50:526–38. 38546855 10.1007/s00134-024-07381-z

[10] Carugati M, Aliberti S, Reyes LF, Franco Sadud R, Irfan M, Prat C, et al. Microbiological testing of adults hospitalised with community-acquired pneumonia: an international study. ERJ Open Res. 2018;4(4). 10.1183/23120541.00096-2018 PMC6174282 30474036

[11] Torres A, Cilloniz C, Niederman MS, Menendez R, Chalmers JD, Wunderink RG, et al. Pneumonia. Nat Rev Dis Primers. 2021;7(1):25. 33833230 10.1038/s41572-021-00259-0

[12] Committee for the Japanese Respiratory Society Guidelines in Management of R. Definition and pathophysiology of hospital-acquired pneumonia. Respirology. 2004;9 Suppl 1:S3–5. 14989723 10.1111/j.1440-1843.2004.00542.x

[13] Shi T, Denouel A, Tietjen AK, Lee JW, Falsey AR, Demont C, et al. Global and regional burden of hospital admissions for pneumonia in older adults: a systematic review and meta-analysis. J Infect Dis. 2020;222(Suppl 7):S570–6. 30849172 10.1093/infdis/jiz053

[14] Monegro AF, Muppidi V, Regunath H. Hospital-acquired infections. Treasure Island: StatPearls; 2023. 28722887

[15] Metlay JP, Waterer GW, Long AC, Anzueto A, Brozek J, Crothers K, et al. Diagnosis and treatment of adults with community-acquired pneumonia. An Official Clinical Practice Guideline of the American Thoracic Society and Infectious Diseases Society of America. Am J Respir Crit Care Med. 2019;200(7):e45–67. 31573350 10.1164/rccm.201908-1581ST PMC6812437

[16] Barlow G, Nathwani D, Myers E, Sullivan F, Stevens N, Duffy R, et al. Identifying barriers to the rapid administration of appropriate antibiotics in community-acquired pneumonia. J Antimicrob Chemother. 2008;61(2):442–51. 18065412 10.1093/jac/dkm462

[17] Chavez MA, Shams N, Ellington LE, Naithani N, Gilman RH, Steinhoff MC, et al. Lung ultrasound for the diagnosis of pneumonia in adults: a systematic review and meta-analysis. Respir Res. 2014;15(1):50. 24758612 10.1186/1465-9921-15-50 PMC4005846

[18] Demi L, Wolfram F, Klersy C, De Silvestri A, Ferretti VV, Muller M, et al. New international guidelines and consensus on the use of lung ultrasound. J Ultrasound Med. 2023;42(2):309–44. 35993596 10.1002/jum.16088 PMC10086956

[19] Rocca E, Zanza C, Longhitano Y, Piccolella F, Romenskaya T, Racca F, et al. Lung ultrasound in critical care and emergency medicine: clinical review. Adv Respir Med. 2023;91(3):203–23. 37218800 10.3390/arm91030017 PMC10204578

[20] Vincent JL, Brealey D, Libert N, Abidi NE, O’Dwyer M, Zacharowski K, et al. Rapid diagnosis of infection in the critically ill, a multicenter study of molecular detection in bloodstream infections, pneumonia, and sterile site infections. Crit Care Med. 2015;43(11):2283–91. 26327198 10.1097/CCM.0000000000001249 PMC4603364

[21] Dureau AF, Duclos G, Antonini F, Boumaza D, Cassir N, Alingrin J, et al. Rapid diagnostic test and use of antibiotic against methicillin-resistant Staphylococcus aureus in adult intensive care unit. Eur J Clin Microbiol Infect Dis. 2017;36(2):267–72. 27714594 10.1007/s10096-016-2795-5

[22] Stafylaki D, Maraki S, Vaporidi K, Georgopoulos D, Kontoyiannis DP, Kofteridis DP, et al. Impact of molecular syndromic diagnosis of severe pneumonia in the management of critically ill patients. Microbiol Spectr. 2022;10(5):e0161622. 36154180 10.1128/spectrum.01616-22 PMC9603977

[23] Peiffer-Smadja N, Bouadma L, Mathy V, Allouche K, Patrier J, Reboul M, et al. Performance and impact of a multiplex PCR in ICU patients with ventilator-associated pneumonia or ventilated hospital-acquired pneumonia. Crit Care. 2020;24(1):366. 32560662 10.1186/s13054-020-03067-2 PMC7303941

[24] Conway Morris A, Bos LDJ, Nseir S. Molecular diagnostics in severe pneumonia: A new dawn or false promise? Intensive Care Med. 2022;48(6):740–2. 35552790 10.1007/s00134-022-06722-0

[25] Barberan J, Restrepo R, Cardinal-Fernandez P. Community-acquired pneumonia: similarities and differences between European and American guidelines—a narrative review. Rev Esp Quimioter. 2021;34(2):72–80. 33291864 10.37201/req/114.2020 PMC8019462

[26] Jain S, Self WH, Wunderink RG, Fakhran S, Balk R, Bramley AM, et al. Community-acquired pneumonia requiring hospitalization among U.S. adults. N Engl J Med. 2015;373(5):415–27. 26172429 10.1056/NEJMoa1500245 PMC4728150

[27] Charles PG, Whitby M, Fuller AJ, Stirling R, Wright AA, Korman TM, et al. The etiology of community-acquired pneumonia in Australia: why penicillin plus doxycycline or a macrolide is the most appropriate therapy. Clin Infect Dis. 2008;46(10):1513–21. 18419484 10.1086/586749

[28] Luyt CE, Hekimian G, Koulenti D, Chastre J. Microbial cause of ICU-acquired pneumonia: hospital-acquired pneumonia versus ventilator-associated pneumonia. Curr Opin Crit Care. 2018;24(5):332–8. 30036192 10.1097/MCC.0000000000000526

[29] Torres A, Lee N, Cilloniz C, Vila J, Van der Eerden M. Laboratory diagnosis of pneumonia in the molecular age. Eur Respir J. 2016;48(6):1764–78. 27811073 10.1183/13993003.01144-2016

[30] Douglas IS. New diagnostic methods for pneumonia in the ICU. Curr Opin Infect Dis. 2016;29(2):197–204. 26859725 10.1097/QCO.0000000000000249

[31] Zar HJ, Madhi SA, Aston SJ, Gordon SB. Pneumonia in low and middle income countries: progress and challenges. Thorax. 2013;68(11):1052–6. 23956020 10.1136/thoraxjnl-2013-204247 PMC3960724

[32] Aston SJ. Pneumonia in the developing world: characteristic features and approach to management. Respirology. 2017;22(7):1276–87. 28681972 10.1111/resp.13112

[33] Koulenti D, Arvaniti K, Judd M, Lalos N, Tjoeng I, Xu E, et al. Ventilator-associated tracheobronchitis: To treat or not to treat? Antibiotics (Basel). 2020;9(2):51. 32023886 10.3390/antibiotics9020051 PMC7168312

[34] Hosmer DW Jr, Lemeshow S, Sturdivant RX. Applied logistic regression. Wiley; 2013.

[35] Sharma A, Minh Duc NT, Luu Lam Thang T, Nam NH, Ng SJ, Abbas KS, et al. A consensus-based checklist for reporting of survey studies (CROSS). J Gen Intern Med. 2021;36(10):3179–87. 33886027 10.1007/s11606-021-06737-1 PMC8481359

[36] Meyer KC, Raghu G, Baughman RP, Brown KK, Costabel U, du Bois RM, et al. An official American Thoracic Society clinical practice guideline: the clinical utility of bronchoalveolar lavage cellular analysis in interstitial lung disease. Am J Respir Crit Care Med. 2012;185(9):1004–14. 22550210 10.1164/rccm.201202-0320ST

[37] Browne E, Hellyer TP, Baudouin SV, Conway Morris A, Linnett V, McAuley DF, et al. A national survey of the diagnosis and management of suspected ventilator-associated pneumonia. BMJ Open Respir Res. 2014;1(1):e000066. 25553248 10.1136/bmjresp-2014-000066 PMC4275666

[38] Mandell LA, Wunderink RG, Anzueto A, Bartlett JG, Campbell GD, Dean NC, et al. Infectious Diseases Society of America/American Thoracic Society consensus guidelines on the management of community-acquired pneumonia in adults. Clin Infect Dis. 2007;44 Suppl 2(Suppl 2):S27-72. 17278083 10.1086/511159 PMC7107997

[39] Lim WS, Baudouin SV, George RC, Hill AT, Jamieson C, Le Jeune I, et al. BTS guidelines for the management of community acquired pneumonia in adults: update 2009. Thorax. 2009;64 Suppl 3:iii1-55. 19783532 10.1136/thx.2009.121434

[40] Plachouras D, Lepape A, Suetens C. ECDC definitions and methods for the surveillance of healthcare-associated infections in intensive care units. Intensive Care Med. 2018;44(12):2216–8. 29797028 10.1007/s00134-018-5113-0 PMC6280825

[41] Masterton RG, Galloway A, French G, Street M, Armstrong J, Brown E, et al. Guidelines for the management of hospital-acquired pneumonia in the UK: report of the working party on hospital-acquired pneumonia of the British Society for Antimicrobial Chemotherapy. J Antimicrob Chemother. 2008;62(1):5–34. 18445577 10.1093/jac/dkn162 PMC7110234

[42] Flateau C, Le Bel J, Tubiana S, Blanc FX, Choquet C, Rammaert B, et al. High heterogeneity in community-acquired pneumonia inclusion criteria: Does this impact on the validity of the results of randomized controlled trials? BMC Infect Dis. 2018;18(1):607. 30509278 10.1186/s12879-018-3515-9 PMC6276130

[43] Fally M, Haseeb F, Kouta A, Hansel J, Robey RC, Williams T, et al. Unravelling the complexity of ventilator-associated pneumonia: a systematic methodological literature review of diagnostic criteria and definitions used in clinical research. Crit Care. 2024;28(1):214. 38956655 10.1186/s13054-024-04991-3 PMC11221085

[44] Miller JM, Binnicker MJ, Campbell S, Carroll KC, Chapin KC, Gilligan PH, et al. A guide to utilization of the microbiology laboratory for diagnosis of infectious diseases: 2018 update by the Infectious Diseases Society of America and the American Society for Microbiology. Clin Infect Dis. 2018;67(6):e1–94. 29955859 10.1093/cid/ciy381 PMC7108105

[45] Bustos IG, Martin-Loeches I, Acosta-Gonzalez A, Chotirmall SH, Dickson RP, Reyes LF. Exploring the complex relationship between the lung microbiome and ventilator-associated pneumonia. Expert Rev Respir Med. 2023;17(10):889–901. 37872770 10.1080/17476348.2023.2273424

[46] Meduri GU, Mauldin GL, Wunderink RG, Leeper KV Jr, Jones CB, Tolley E, et al. Causes of fever and pulmonary densities in patients with clinical manifestations of ventilator-associated pneumonia. Chest. 1994;106(1):221–35. 8020275 10.1378/chest.106.1.221

[47] Morris AC, Kefala K, Simpson AJ, Wilkinson TS, Everingham K, Kerslake D, et al. Evaluation of the effect of diagnostic methodology on the reported incidence of ventilator-associated pneumonia. Thorax. 2009;64(6):516–22. 19213771 10.1136/thx.2008.110239

[48] Ewig S, Torres A, El-Ebiary M, Fabregas N, Hernandez C, Gonzalez J, et al. Bacterial colonization patterns in mechanically ventilated patients with traumatic and medical head injury. Incidence, risk factors, and association with ventilator-associated pneumonia. Am J Respir Crit Care Med. 1999;159(1):188–98. 9872838 10.1164/ajrccm.159.1.9803097

[49] Luyt CE, Hekimian G, Bonnet I, Brechot N, Schmidt M, Robert J, et al. Usefulness of point-of-care multiplex PCR to rapidly identify pathogens responsible for ventilator-associated pneumonia and their resistance to antibiotics: an observational study. Crit Care. 2020;24(1):378. 32586347 10.1186/s13054-020-03102-2 PMC7316635

[50] Evans SE, Jennerich AL, Azar MM, Cao B, Crothers K, Dickson RP, et al. Nucleic acid-based testing for noninfluenza viral pathogens in adults with suspected community-acquired pneumonia. An Official American Thoracic Society Clinical Practice Guideline. Am J Respir Crit Care Med. 2021;203(9):1070–87. 33929301 10.1164/rccm.202102-0498ST PMC8314899

[51] Hellyer TP, Morris AC, McAuley DF, Walsh TS, Anderson NH, Singh S, et al. Diagnostic accuracy of pulmonary host inflammatory mediators in the exclusion of ventilator-acquired pneumonia. Thorax. 2015;70(1):41–7. 25298325 10.1136/thoraxjnl-2014-205766 PMC4992819

[52] Bougle A, Tuffet S, Federici L, Leone M, Monsel A, Dessalle T, et al. Comparison of 8 versus 15 days of antibiotic therapy for Pseudomonas aeruginosa ventilator-associated pneumonia in adults: a randomized, controlled, open-label trial. Intensive Care Med. 2022;48(7):841–9. 35552788 10.1007/s00134-022-06690-5

[53] Yi SH, Hatfield KM, Baggs J, Hicks LA, Srinivasan A, Reddy S, et al. Duration of antibiotic use among adults with uncomplicated community-acquired pneumonia requiring hospitalization in the United States. Clin Infect Dis. 2018;66(9):1333–41. 29126268 10.1093/cid/cix986 PMC6474781

[54] Gordon K, Stevens R, Westley B, Bulkow L. Impact of an antimicrobial stewardship program on outcomes in patients with community-acquired pneumonia admitted to a tertiary community hospital. Am J Health Syst Pharm. 2018;75(11 Supplement 2):S42–50. 29802178 10.2146/ajhp170360 PMC11376219

[55] Leo F, Bannehr M, Valenta S, Lippeck M, Pachl S, Steib-Bauert M, et al. Impact of a computerized physician order entry (CPOE)-based antibiotic stewardship intervention on the treatment duration for pneumonia and COPD exacerbations. Respir Med. 2021;186:106546. 34332265 10.1016/j.rmed.2021.106546

[56] Kurtzhalts KE, Sellick JA Jr, Ruh CA, Carbo JF, Ott MC, Mergenhagen KA. Impact of antimicrobial stewardship on outcomes in hospitalized veterans with pneumonia. Clin Ther. 2016;38(7):1750–8. 27349712 10.1016/j.clinthera.2016.06.004

[57] Waagsbo B, Tranung M, Damas JK, Heggelund L. Antimicrobial therapy of community-acquired pneumonia during stewardship efforts and a coronavirus pandemic: an observational study. BMC Pulm Med. 2022;22(1):379. 36242006 10.1186/s12890-022-02178-6 PMC9569007

[58] Foolad F, Huang AM, Nguyen CT, Colyer L, Lim M, Grieger J, et al. A multicentre stewardship initiative to decrease excessive duration of antibiotic therapy for the treatment of community-acquired pneumonia. J Antimicrob Chemother. 2018;73(5):1402–7. 29462306 10.1093/jac/dky021

[59] Lim WS, Smith DL, Wise MP, Welham SA. British Thoracic Society community acquired pneumonia guideline and the NICE pneumonia guideline: how they fit together. BMJ Open Respir Res. 2015;2(1):e000091. 26019876 10.1136/bmjresp-2015-000091 PMC4442154

[60] Kett DH, Cano E, Quartin AA, Mangino JE, Zervos MJ, Peyrani P, et al. Implementation of guidelines for management of possible multidrug-resistant pneumonia in intensive care: an observational, multicentre cohort study. Lancet Infect Dis. 2011;11(3):181–9. 21256086 10.1016/S1473-3099(10)70314-5

