# Correction: D-PRISM: a global survey-based study to assess diagnostic and treatment approaches in pneumonia managed in intensive care

**DOI:** 10.1186/s13054-025-05610-5

**Published:** 2025-10-16

**Authors:** Luis Felipe Reyes, Cristian C. Serrano‑Mayorga, Zhongheng Zhang, Isabela Tsuji, Gennaro De Pascale, Valeria Enciso Prieto, Mervyn Mer, Elyce Sheehan, Prashant Nasa, Goran Zangana, Kostoula Arvaniti, Alexis Tabah, Gentle Sunder Shrestha, Hendrik Bracht, Arie Zainul Fatoni, Khalid Abidi, Helmi bin Sulaiman, Vandana Kalwaje Eshwara, Liesbet De Bus, Yoshiro Hayashi, Pervin Korkmaz, Ali Ait Hssain, Niccolo Buetti, Qing Yuan Goh, Arthur Kwizera, Despoina Koulenti, Nathan D. Nielsen, Pedro Povoa, Otavio Ranzani, Jordi Rello, Andrew Conway Morris

**Affiliations:** 1 Unisabana Center for Translational Science, School of Medicine, Universidad de La Sabana, Chia, Colombia; 2 Clinica Universidad de La Sabana, Chia, Colombia; 3 PhD Biosciences Program, Engineering School, Universidad de La Sabana, Chia, Colombia; 4 Pandemic Sciences Institute, University of Oxford, Oxford, UK; 5 Department of Emergency Medicine, Sir Run Run Shaw Hospital, Zhejiang University School of Medicine, Hangzhou, China; 6 Hospital Israelita Albert Einstein, Sao Paulo, Brazil; 7 Dipartimento di Scienze Biotecnologiche di Base, Cliniche Intensivologiche e Perioperatorie, Universita Cattolica del Sacro Cuore, Rome, Italy; 8 Dipartimento di Scienze dell’Emergenza, Anestesiologiche e della Rianimazione, Fondazione Policlinico Universitario A. Gemelli IRCCS, Rome, Italy; 9 Divisions of Critical Care and Pulmonology, Department of Medicine, Charlotte Maxeke Johannesburg Academic Hospital and Faculty of Health Sciences, University of the Witwatersrand, Johannesburg, South Africa; 10 Division of Pulmonary, Critical Care and Sleep Medicine, University of New Mexico School of Medicine, Albuquerque, USA; 11 Critical Care Medicine NMC Specialty Hospital Dubai, Dubai, UAE; 12 Internal Medicine, College of Medicine and Health Sciences, Al Ain, UAE; 13 Department of Acute and General Medicine, Royal Infirmary of Edinburgh, Edinburgh, Scotland, UK; 14 Intensive Care Medicine, Papageorgiou Hospital, Thessaloniki, Greece; 15 Queensland University of Technology, Brisbane, QLD Australia; 16 Intensive Care Unit, Redcliffe Hospital, Metro North Hospital and Health Services, Brisbane, QLD Australia; 17 Faculty of Medicine, The University of Queensland, Brisbane, QLD Australia; 18 Department of Critical Care Medicine, Tribhuvan University Teaching Hospital, Maharajgunj, Kathmandu, Nepal; 19 Department of Anesthesiology, Intensive Care, Emergency Medicine, Transfusion Medicine, and Pain Therapy, Protestant Hospital of the Bethel Foundation, University Hospital of Bielefeld, Campus Bielefeld-Bethel, Bielefeld, Germany; 20 Department of Anesthesiology and Intensive Therapy, Saiful Anwar General Hospital ‑ Faculty of Medicine, Brawijaya University, Malang, East Java, Indonesia; 21 Ibn Sina University Hospital, Faculty of Medicine and Pharmacy, Mohammed V University, Rabat, Morocco; 22 Infectious Diseases Unit, Department of Medicine, Faculty of Medicine, University of Malaya, Kuala Lumpur, Malaysia; 23 Department of Microbiology Kasturba Medical College, Manipal Manipal Academy of Higher Education, Manipal, Karnataka India; 24 Department of Intensive Care Medicine, Ghent University Hospital, Ghent, Belgium; 25 Department of Internal Medicine and Pediatrics, Faculty of Medicine and Health Sciences, Ghent University, Ghent, Belgium; 26 Department of Intensive Care Medicine, Kameda Medical Center, Kamogawa, Japan; 27 Pulmonary Disease Department, Ege University School of Medicine, Izmir, Turkey; 28 Medical Intensive Care Unit, Hamad General Hospital, Doha, Qatar; 29 Infection Control Program, Geneva University Hospitals and Faculty of Medicine, World Health Organization Collaborating Centre, Geneva, Switzerland; 30 IAME UMR 1137, INSERM, Université Paris-Cité, Paris, France; 31 Division of Anaesthesiology and Perioperative Medicine, Department of Surgical Intensive Care, Singapore General Hospital, Singapore, Singapore; 32 Department of Anaesthesia, Makerere University, Kampala, Uganda; 33 Department of Critical Care, King’s College Hospital NHS Foundation Trust, London, UK; 34 Antibiotic Optimisation Group, UQ Centre for Clinical Research, Faculty of Medicine, The University of Queensland, Brisbane, Australia; 35 Section of Transfusion Medicine and Therapeutic Pathology, University of New Mexico School of Medicine, Albuquerque, USA; 36 Faculdade de Ciências Médicas, NOVA Medical School, NOVA University of Lisbon, Lisbon, Portugal; 37 Center for Clinical Epidemiology and Research Unit of Clinical Epidemiology, OUH Odense University Hospital, Odense, Denmark; 38 Department of Intensive Care, Hospital de São Francisco Xavier, ULSLO, Lisbon, Portugal; 39 Barcelona Institute for Global Health, ISGlobal, Hospital Clinic-Universitatde Barcelona, Barcelona, Spain; 40 Pulmonary Division, Heart Institute (InCor), Hospital das Clinicas HCFMUSP, Faculdade de Medicina, Universidade de Sao Paulo, São Paulo, Brazil; 41 Vall d’Hebron Institute of Research, Barcelona, Spain; 42 Pormation, Recherche & Evaluation (FOREVA), CHU Nimes, Nimes, France; 43 Centro de Investigación Biomédica en Red (CIBERES), Instituto de Salud Carlos III, Madrid, Spain; 44 Division of Perioperative, Acute, Critical Care and Emergency Medicine, Department of Medicine, University of Cambridge, Level 4, Addenbrooke’s Hospital, Hills Road, Cambridge, UK; 45 Division of Immunology, Department of Pathology, University of Cambridge, Cambridge, UK; 46 John V Farman Intensive Care Unit, Addenbrooke’s Hospital, Cambridge, UK

**Correction: Critical Care (2024) 28:381**

10.1186/s13054-024-05180-y

Following the publication of the original article [1], the authors identified an error in the author name of Kostoula Arvaniti and that the equal contribution was missing for Luis Felipe Reyes and Cristian C. Serrano‑Mayorga.

The incorrect author name is:

Kostoula Avanti.

The correct author name is:

Kostoula Arvaniti.

The author group has been updated in this correction article and the original article [1] has been corrected.
