# Supplementary material for: D-PRISM: a global survey-based study to assess diagnostic and treatment approaches in pneumonia managed in intensive care
Source: Crit Care. 2024 Nov 22;28:381. doi: 10.1186/s13054-024-05180-y (PMC11585090; doi:10.1186/s13054-024-05180-y)
Supplement: Supplementary file 1 — Additional file 1. [file 13054_2024_5180_MOESM1_ESM.docx]

**D-PRISM:** D-PRISM: A Global Survey-based Study to Assess Diagnostic and Treatment Approaches in Pneumonia Managed in Intensive Care.

**AUTHORS:** Luis Felipe Reyes, Cristian C Serrano-Mayorga, Zhongheng Zhang, Isabela Tsuji, Gennaro De Pascale, Valeria Enciso Prieto, Mervyn Mer, Elyce Sheehan, Prashant Nasa, Goran Zangana, Kostoula Avanti, Alexis Tabah, Gentle Shrestha, Hendrik Bracht, Arie Zainul, Khalid Abidi, Helmi bin Sulaiman, Vandana Kalwaje Eshwara, Liesbet De Bus, Yoshiro Hayashi, Pervin Korkmaz, Ali Ait Hssain, Niccolò Buetti, Qing Yuan Goh, Arthur Kwizera, Despoina Koulenti, Nathan D. Nielsen, Pedro Povoa, Otavio Ranzani, Jordi Rello, Andrew Conway Morris for the D-PRISM investigators.

**Tables**

**Table S1.** Distribution between type of health care centre and income categories.

| **Type of Health Care Centre / Income category** | **Total Cohort n =1296** | **High-Income n =640** | **Upper-Middle Income n = 448** | **Low and Lower Middle-Income n =208** |
| --- | --- | --- | --- | --- |
| **Community District**  **Hospital** | 338 (26.0%) | 188 (29.3%) | 95 (21.2%) | 55 (26.4%) |
| **Remote and rural Hospital** | 41 (3.1%) | 22 (3.4%) | 8 (1.8%) | 11 (5.2%) |
| **Teaching Hospital** | 913 (70.4%) | 428 (66.8%) | 343 (76.6%) | 208 (68.3%) |
| **Not Answer** | 4 (0.3%) | 2 (0.3%) | 2 (0.4%) | 0 (0%) |

**Tables S2.** Respondents' assessment rates for diagnostic criteria classified by The World Bank income classification.

| **Diagnostic criteria** | **CAP** | | | | **HAP** | | | | **VAP** | | | |
| --- | --- | --- | --- | --- | --- | --- | --- | --- | --- | --- | --- | --- |
|  | **All Cohort n=1296** | **High- income n=640** | **Upper-middle income n=448** | **Low and Lower-middle income n=208** | **All Cohort n=1296** | **High- income n=640** | **Upper-middle income n=448** | **Low and Lower-middle income n=208** | **All Cohort n=1296** | **High- income n=640** | **Upper-middle income n=448** | **Low and Lower-middle income n=208** |
| **Clinical presentation | 1235 (95%) | 618 (96%) | 426 (95%) | 191 (92%) | 1234 (95%) | 614 (95%) | 430 (96%) | 191 (92%) | 1218 (94%) | 595 (93%) | 431(96%) | 193 (93%) |
| **Radiological criteria** | | | | | | | | | | | | |
| **Chest X-ray or CT scan | 1257 (97%) | 629 (98%) | 434 (96%) | 194 (93%) | 1259 (97%) | 632 (98%) | 435 (97%) | 193 (93%) | 1244 (96%) | 624 (97%) | 426 (95%) | 195 (94%) |
| **Lung ultrasound | 379 (29%) | 163 (25%) | 130 (29%) | 67 (32%) | 405 (31%) | 173 (27%) | 146 (32%) | 87 (42%) | 443 (34%) | 199 (31%) | 157 (35%) | 88 (42%) |
| **Clinical and radiological criteria** | | | | | | | | | | | | |
| *Clinical presentation and Chest X-ray or CT scan | 845 (65%) | 448 (70%) | 281 (63%) | 116 (56%) | 747 (58%) | 387 (60%) | 257 (57%) | 103 (46%) | 742 (57%) | 376 (59%) | 245 (55%) | 55 (26%) |
| *Clinical presentation and lung ultrasound | 362 (28%) | 41 (6%) | 47 (10%) | 26 (12%) | 115 (9%) | 43 (7%) | 45 (10%) | 27 (13%) | 151 (12%) | 58 (9%) | 60 (13%) | 33 (16%) |
| Clinical presentation included the following signs and symptoms: positive findings on auscultation such as bronchi, crepitations, wheeze, breathlessness, purulent sputum, impaired oxygenation or ventilation; Radiological findings included: CXR or C.T. showing lobar infiltration or bronchogram or diffuse or patchy shadowing; Sonological findings include USS showing consolidation or bronchogram.  * Frequencies reported only include the "always" response  ** Frequencies reported the combination of "always" and "mostly" response. | | | | | | | | | | | | |

**Table S3.** Relation between Antibiotic Time-out and regimen.

| **n=1296** | **VAP Antibiotic Time-Out** | | | **HAP Antibiotic Time-Out** | | |
| --- | --- | --- | --- | --- | --- | --- |
|  | **Yes n= 693 (53.4%)** | **No n=597 (46.0%)** | **N/A n= 6 (0.5%)** | **Yes = 671 (51.7%)** | **No n=612 (47.2%)** | **N/A n=13 (1%)** |
| Monotherapy for all patients | 100 (14.4%) | 63 (10.5%) | 0 (0%) | 116 (17.2%) | 91 (14.9%) | 2 (15.4%) |
| Monotherapy for low risk of resistant organisms | 247 (35.6%) | 263 (44.0%) | 1 (16.6%) | 283 (42.1%) | 285 (46.6%) | 4 (30.8%) |
| Dual therapy including coverage for resistant organisms (eg MRSA/MDR Pseudomonas) for all patients | 346 (49.9%) | 269 (45.0%) | 1 (16.6%) | 271 (40.3%) | 235 (38.4%) | 4 (30.8%) |
| N/A | 0 (0%) | 2 (0.3%) | 4 (66.6%) | 1 (0.1%) | 1 (0.1%) | 3 (23.1%) |

**Table S4.** Univariable and multivariable regression for factors associated with antimicrobial treatment less than 7 days in CAP.

| **CAP Regression for Treatment < 7 days** | | | | |
| --- | --- | --- | --- | --- |
| **Factor** | **Univariable** | | **Multivariable** | |
|  | **p-Value** | **OR (95% IC)** | **p-Value** | **OR (95% IC)** |
| Intensivist | 0.004 | 1.17 (1.20-2.43) | 0.04 | 1.47 (1.00-2.15) |
| **Post Graduate ICU Experience** | | | | |
| <5 years | 0.85 | 0.95 (0.67-1.36) |  |  |
| 5- 10 years | 0.92 | 1.03 (0.70-1.51) |  |  |
| 10 years | 1.00 | 1.00 (0.72-1.38) |  |  |
| **Hospital Characteristics** | | | | |
| University/Teaching Hospital | 0.18 | 1.27 (0.90-1.78) | 0.54 | 1.11 (0.78-1.59) |
| Community Distric Hospital | 0.40 | 0.85 (0.60-1.22) |  |  |
| Remote and rural Hospital | 0.47 | 0.70 (0.30-1.63) |  |  |
| **Protocol of hospital** | | | | |
| Antimicrobial Stewardship Program | <0.001 | 2.13 (1.50-3.02) | <0.001 | 1.93 (1.33-2.78) |
| Local Antimicrobial Guidelines | 0.05 | 1.45 (1.00-2.10) |  |  |
| **Income Level** | | | | |
| High-Income | 0.018 | 1.48 (1.07-2.05) | 0.65 | 1.08 (0.75-1.55) |
| Upper-middle income | 0.07 | 0.74 (0.53-1.02) |  |  |
| Lower-middle income | 0.49 | 0.85 (0.55-1.31) |  |  |
| Low Income | 0.24 | 0.38 (0.07-2.02) |  |  |
| **Microbiological Test** | | | | |
| Respiratory PCR Multiplex | 0.31 | 0.78 (0.51-1.19) |  |  |
| **Diagnosis** | | | | |
| Clinical presentation and Chest X-ray or CT scan | 0.016 | 1.20 (0.87-1.65) | 0.034 | 1.43 (1.02-2.00) |
| **Antimicrobial** | | | | |
| Dual Therapy with macrolide | <0.001 | 1.42 (1.01-1.99) | <0.001 | 1.78 (1.28-2.47) |
| Dual Therapy with non-macrolide | <0.001 | 1.75 (0.98-3.11) |  |  |

**Table S5.** Univariable and multivariable regression for factors associated with antimicrobial treatment less than 7 days in HAP.

| **HAP Regression for Treatment < 7 days** | | | | |
| --- | --- | --- | --- | --- |
|  | **Univariable** | | **Multivariable** | |
|  | **p-Value** | **OR (95% IC)** | **p-Value** | **OR (95% IC)** |
| Intensivist | <0.001 | 1.79 (1.38-2.33) | 0.01 | 1.46 (1.09-1.96) |
| **Post Graduate ICU Experience** | | | | |
| <5 years | 0.19 | 0.84 (0.66-1.08) |  |  |
| 5- 10 years | 0.28 | 1.16 (0.89-1.51) |  |  |
| 10 years | 0.69 | 1.04 (0.83-1.30) |  |  |
| **Hospital Characteristics** | | | | |
| University/Teaching Hospital | 0.49 | 1.09 (0.86-1.39) |  |  |
| Community Distric Hospital | 1.00 | 0.99 (0.77-1.28) |  |  |
| Remote and rural Hospital | 0.19 | 0.64 (0.34-1.20) |  |  |
| General ICU | 0.22 | 1.17 (0.90-1.53) |  |  |
| Medical ICU | 0.05 | 0.71 (0.51-1.00) |  |  |
| Cardiac ICU | 0.72 | 0.86 (0.43-1.72) |  |  |
| Surgical ICU | 0.39 | 1.25 (0.76-2.05) |  |  |
| Neurological ICU | 1.00 | 0.96 (0.42-2.18) |  |  |
| **Protocol Hospital** | | | | |
| Antimicrobial Stewardship Program | <0.001 | 2.12 (1.61-2.77) | <0.001 | 1.79 (1.35-2.39) |
| Local Antimicrobial Guidelines | 0.006 | 1.47 (1.12-1.93) |  |  |
| **Income Level** | | | | |
| High-Income | <0.001 | 1.85 (1.47-2.31) | 0.004 | 1.45 (1.13-1.87) |
| Upper-middle income | <0.001 | 0.64 (0.51-0.81) |  |  |
| Lower-middle income | 0.029 | 0.71 (0.52-0.96) |  |  |
| Low Income | 0.128 | 0.27 (0.05-1.41) | 0.51 | 0.56 (0.10-3.16) |
| **Microbiological Test** | | | | |
| Respiratory PCR Multiplex | 0.27 | 1.15 (0.88-1.51) |  |  |
| **Diagnosis** | | | | |
| Clinical presentation and Chest X-ray or CT scan | 0.08 | 1.21 (0.97-1.52) | 0.42 | 1.10 (0.86-1.40) |
| **Antimicrobial** | | | | |
| Antibiotic Time-out | 0.002 | 1.42 (1.14-1.78) | 0.002 | 1.46 (1.15-1.85) |
| Dual Therapy MRSA,MDR, *Pseudomonas* | <0.001 | 0.50 (0.40-0.63) | 0.003 | 0.68 (0.53-0.87) |
| Dual Therapy MRSA,MDR, *Pseudomonas* only for High Risk Patients | 0.53 | 1.07 (0.86-1.34) |  |  |
| Monotherapy for all Patients | <0.001 | 3.40 (2.36-4.90) | <0.001 | 2.55 (1.72-3.77) |
| Monotherapy only for Low Risk Patients | 0.53 | 1.07 (0.86-1.34) |  |  |

**Table S6.** Univariable and multivariable regression for factors associated with antimicrobial treatment less than 7 days in VAP.

| **VAP Regression for Treatment < 7 days** | | | | |
| --- | --- | --- | --- | --- |
|  | **Univariable** | | **Multivariable** | |
|  | **p-Value** | **OR (95% IC)** | **p-Value** | **OR (95% IC)** |
| Intensivist | <0.001 | 1.64 (1.26-2.13) | 0.07 | 1.30 (0.97-1.75) |
| **Post Graduate ICU Experience** | | | | |
| <5 years | 0.18 | 0.84 (0.66-1.07) |  |  |
| 5- 10 years | 0.29 | 1.14 (0.88-1.48) |  |  |
| 10 years | 0.65 | 1.05 (0.84-1.30) |  |  |
| **Hospital Characteristics** | | | | |
| University/Teaching Hospital | 0.39 | 1.11 (0.87-1.41) |  |  |
| Community Distric Hospital | 0.75 | 0.95 (0.74-1.22) |  |  |
| Remote and rural Hospital | 0.34 | 0.73 (0.39-1.36) |  |  |
| General ICU | 0.94 | 0.98 (0.76-1.28) |  |  |
| Medical ICU | 0.15 | 0.77 (0.55-1.08) |  |  |
| Cardiac ICU | 0.86 | 0.94 (0.47-1.86) |  |  |
| Surgical ICU | 0.05 | 1.65 (1.01-2.69) | 0.26 | 1.34 (0.79-2.28) |
| Neurological ICU | 0.54 | 1.32 (0.58-3.01) |  |  |
| **Protocol Hospital** | | | | |
| Antimicrobial Stewardship Program | <0.001 | 2.19 (1.66-2.89) | <0.001 | 1.74 (1.30-2.33) |
| Local Antimicrobial Guidelines | 0.02 | 1.36 (1.04-1.79) |  |  |
| **Income Level** | | | | |
| High-Income | <0.001 | 2.00 (1.60-2.49) | <0.001 | 1.62 (1.26-2.07) |
| Upper-middle income | <0.001 | 0.62 (0.49-0.78) |  |  |
| Lower-middle income | 0.003 | 0.63 (0.46-0.85) |  |  |
| Low Income | 0.27 | 0.37 (0.07-1.94) |  |  |
| **Microbiological Test** | | | | |
| Respiratory PCR Multiplex | 0.12 | 1.23 (0.94-1.59) |  |  |
| **Diagnosis** | | | | |
| Clinical presentation and Chest X-ray or CT scan | 0.64 | 1.05 (0.84-1.32) |  |  |
| **Antimicrobial** | | | | |
| Antibiotic Time-out | 0.001 | 1.43 (1.15-1.78) | <0.001 | 1.46 (1.16-1.85) |
| Dual Therapy MRSA,MDR, Pseudomonas | <0.001 | 0.48 (0.38-0.60) | 0.002 | 0.67 (0.52-0.86) |
| Dual Therapy MRSA,MDR, Pseudomonas only for High Risk Patients | 0.23 | 1.15 (0.92-1.43) |  |  |
| Monotherapy for all Patients | <0.001 | 4.51 (3.01-6.75) | <0.001 | 3.22 (2.09-4.97) |
| Monotherapy only for Low Risk Patients | 0.23 | 1.15 (0.92-1.43) |  |  |

**Table S7.** Comparison of Sample/Test availability according to World Bank income classification

| **Response** | **All Cohort n=1296** | **High-income n=640** | **Upper-middle n= 448** | **Low and Lower middle income n=208** |
| --- | --- | --- | --- | --- |
| **Sputum** | | | | |
| Don't know | 59 (4.6%) | 28 (4.4%) | 17 (3.8%) | 14 (6.7%) |
| N/A | 24 (1.9%) | 8 (1.3%) | 7 (1.6%) | 9 (4.3%) |
| Positive/Negative | 162 (12.5%) | 79 (12.3%) | 54 (12.1%) | 29 (13.9%) |
| Qualitative (small, moderate, heavy growth) | 336 (25.9%) | 213 (33.3%) | 76 (17%) | 47 (22.6%) |
| Quantitative/Semi-quantitative (Colony Forming Unit) | 696 (53.7%) | 309 (48.3%) | 287 (64.1%) | 100 (48.1%) |
| This technique not used in my hospital | 19 (1.5%) | 3 (0.5%) | 7 (1.6%) | 9 (4.3%) |
| **Blind Mini BAL** | | | | |
| Don't know | 149 (11.5%) | 69 (10.8%) | 55 (12.3%) | 25 (12%) |
| N/A | 63 (4.9%) | 21 (3.3%) | 19 (4.2%) | 23 (11.1%) |
| Positive/Negative | 114 (8.8%) | 56 (8.8%) | 35 (7.8%) | 23 (11.1%) |
| Qualitative (small, moderate, heavy growth) | 238 (18.4%) | 142 (22.2%) | 60 (13.4%) | 36 (17.3%) |
| Quantitative/Semi-quantitative (Colony Forming Unit) | 559 (43.1%) | 264 (41.3%) | 219 (48.9%) | 76 (36.5%) |
| This technique not used in my hospital | 173 (13.3%) | 88 (13.8%) | 60 (13.4%) | 25 (12%) |
| **BAL** | | | | |
| Don't know | 107 (8.3%) | 47 (7.3%) | 35 (7.8%) | 25 (12%) |
| N/A | 61 (4.7%) | 17 (2.7%) | 16 (3.6%) | 28 (13.5%) |
| Positive/Negative | 107 (8.3%) | 56 (8.8%) | 39 (8.7%) | 12 (5.8%) |
| Qualitative (small, moderate, heavy growth) | 246 (19%) | 146 (22.8%) | 69 (15.4%) | 31 (14.9%) |
| Quantitative/Semi-quantitative (Colony Forming Unit) | 737 (56.9%) | 373 (58.3%) | 276 (61.6%) | 88 (42.3%) |
| This technique not used in my hospital | 38 (2.9%) | 1 (0.2%) | 13 (2.9%) | 24 (11.5%) |
| **Blood Culture** | | | | |
| Don't know | 43 (3.3%) | 22 (3.4%) | 11 (2.5%) | 10 (4.8%) |
| N/A | 28 (2.2%) | 8 (1.3%) | 6 (1.3%) | 14 (6.7%) |
| Positive/Negative | 382 (29.5%) | 242 (37.8%) | 96 (21.4%) | 44 (21.2%) |
| Qualitative (small, moderate, heavy growth) | 215 (16.6%) | 113 (17.7%) | 53 (11.8%) | 49 (23.6%) |
| Quantitative/Semi-quantitative (Colony Forming Unit) | 619 (47.8%) | 255 (39.8%) | 282 (62.9%) | 82 (39.4%) |
| This technique not used in my hospital | 9 (0.7%) | 0 (0%) | 0 (0%) | 9 (4.3%) |
| N/A : Not Answered ; BAL: Broncho-Alveolar Lavage | | | | |

**Figures**

**Figure S1.** Number of respondents per country


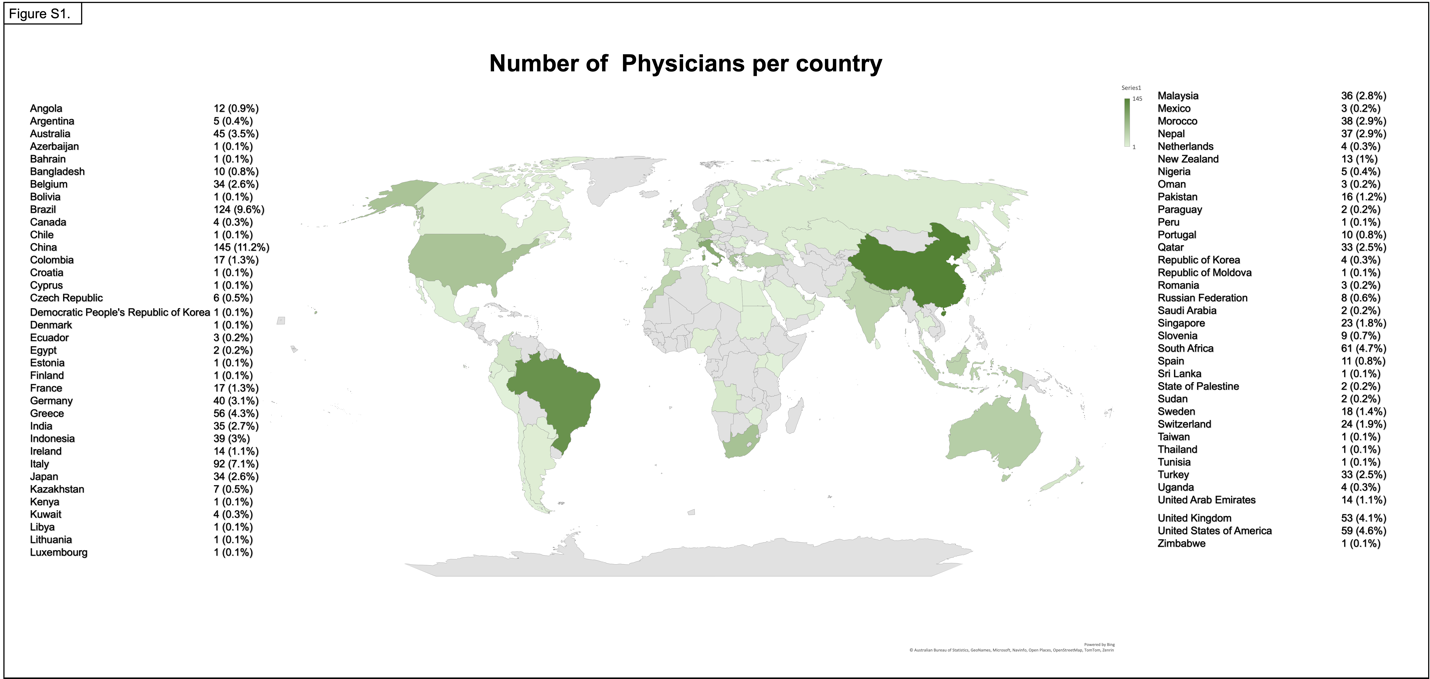


**Figure S2.** World Bank's classification of regions.

**
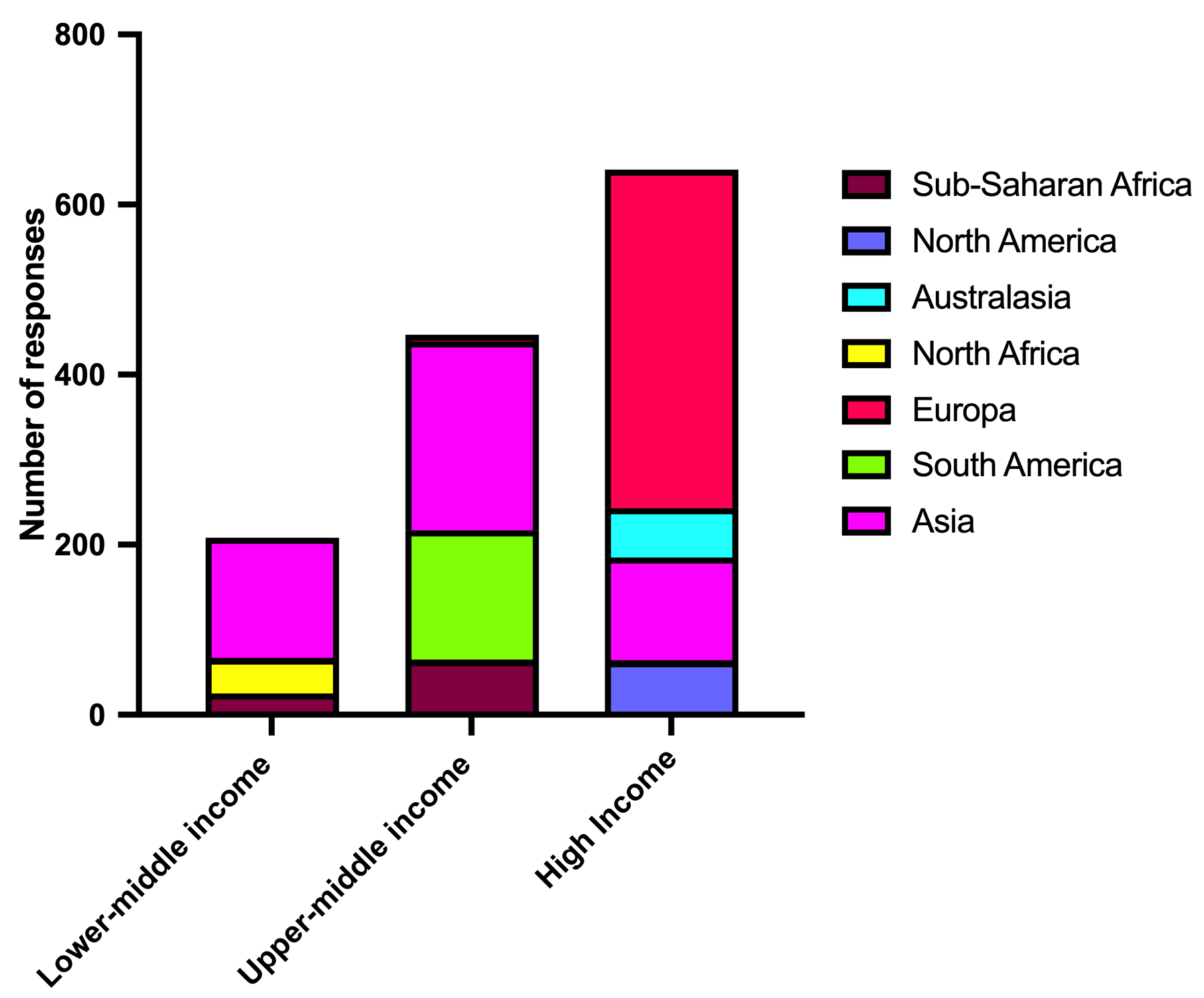
**

**Figure S3.** Forrest Plot of Multivariable analysis for factors associated with antibiotic treatment less than 7 days in **A.** CAP **B.** HAP **C.** VAP

**
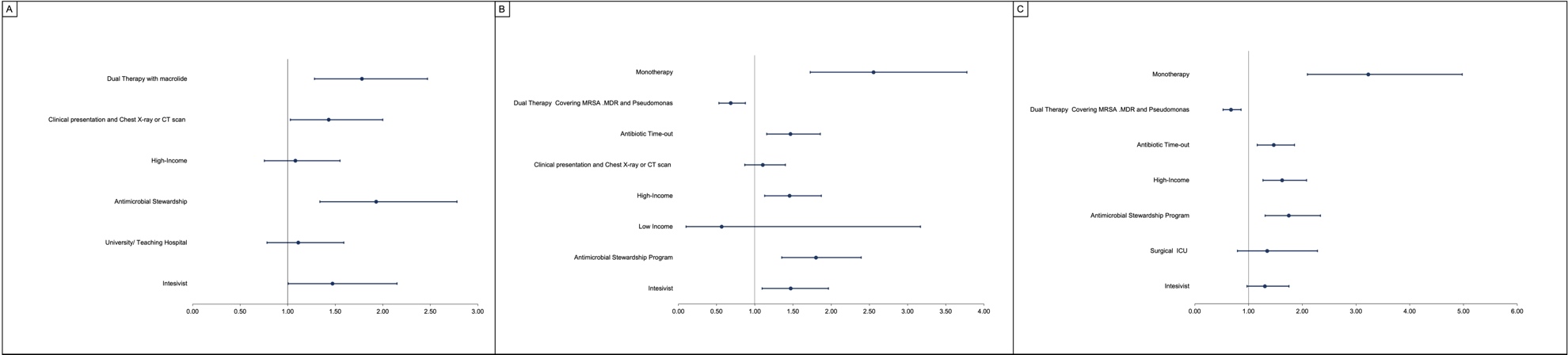
**

**Figure S4.** Multiplex tests were reported by respondents.
