# Supplementary material for: D-PRISM: a global survey-based study to assess diagnostic and treatment approaches in pneumonia managed in intensive care
Source: Crit Care. 2024 Nov 22;28:381. doi: 10.1186/s13054-024-05180-y (PMC11585090; doi:10.1186/s13054-024-05180-y)
Supplement: Supplementary file 2 — Additional file 2. [file 13054_2024_5180_MOESM2_ESM.pdf]

Diagnostic aPpRoaches In Severe pneuMonia - the D-PRISM survey study

Definitions
For the purposes of this survey the following definitions will be used:

1. Community acquired Pneumonia (CAP) : Pneumonia or suspected pneumonia present at hospital admission or manifesting within 48 hours of hospital admission.
2. Hospital Acquired Pneumonia (HAP): Pneumonia or suspected pneumonia not present at hospital admission and developing at least 48 hours after hospital admission (includes Non-ventilated ICU patients)
3. Ventilator Associated Pneumonia (VAP): Pneumonia or suspected pneumonia not present at ICU admission and developing at least 48 hours after initiation of mechanical ventilation (includes patients within 48 hours of extubation).

1. Specialty (please indicate all certified or training in)

- [ ] Intensivist
- [ ] Respiratory Physician
- [ ] Anaesthetist
- [ ] Other (please specify)

____

2. Grade

- ○ Consultant/Specialist
- ○ Trainee/Resident

3. Postgraduate ICU experience (years)

- ○ < 5
- ○ 5-10
- ○ >10

4. In what country do you work?

____

5. Unit Type

- ○ General ICU (mixed medical and surgical)
- ○ Cardiac ICU
- ○ Neuro ICU
- ○ Surgical ICU
- ○ Medical ICU

6. Unit Setting

- ◯ University/ Teaching Hospital
- ◯ Community District Hospital
- ◯ Remote and rural hospital

7. Total number of beds in hospital

[ ]

8. Total number of beds in unit

[ ]

9. Routine management of hematological malignancy/bone marrow transplant/organ transplant (>5 patients from any of these groups/month)?

- ◯ Yes
- ◯ No

10. Do you have guidelines for antibiotic prescribing in severe pneumonia?

- ◯ Yes
- ◯ No

11. Do you have an antimicrobial stewardship programme/ agreed protocol for monitoring use of antibiotics/ involvement of microbiologists/infection diseases experts?

- ◯ Yes
- ◯ No

12. Do you have access to diagnostic bronchoscopy?

- ◯ Yes
- ◯ No

13. If yes to Q12, please state availability

- ◯ 24 hours, 7 days/week
- ◯ Specific hours, every day
- ◯ Specific weekdays only

14. Who provides diagnostic bronchoscopy?

- ◯ Intensivist
- ◯ Pulmonologist from outside unit
- ◯ Other (please specify)

[ ]

15. In units performing own BAL, how would you classify your level of training?

- Formal training
- Informal training
- No training

16. On a scale of 0-10, how would you rate your level of confidence in performing BAL?

17. On average, what is the instilled volume (ml) you use for BAL?

18. What does your lab report for each of the following microbiological samples?

	Quantitative/Semi-quantitative (Colony Forming Unit)	Qualitative (small, moderate, heavy growth)	Positive/Negative	This technique is not used in my hospital
Sputum/Endotracheal aspirates				
blind (non-bronchoscopic) mini-BAL				
Bronchoscopic BAL				
Blood Cultures				
Other body fluids (eg pleural fluid)				

19. Do you use any of the following in the diagnosis of pneumonia (CAP,HAP and/or VAP - see top of survey for definitions of these)?

	Yes	No	Don't know
Streptococcal urinary antigen			
Legionella urinary antigen			
Single organism PCR - if yes, see Q22			
Multiplex Respiratory pathogens- if yes, see Q23			

20. If single organism PCRs are used, please specify the organism(s) (leave blank if not used)

21. Please state which platform you use for multiplex respiratory pathogens (leave blank if not used)

- ○ Biofire Filmarray
- ○ Curetis Unyvero
- ○ Seegene Allplex
- ○ Qiagen RespiFinder
- ○ In-house lab developed test
- ○ Multiplex used but unsure of name
- ○ Other commercial assay (please specify)

[ ]

22. If there are barriers to the use of multiplex respiratory PCR assays in your hospital please list them here (e.g. cost, lack of expertise, difficulty obtaining reagents)?

[ ]

23. In Community Acquired Pneumonia (CAP), how frequently is each of the following diagnostic criteria assessed? Select the most appropriate response:

	Always	Mostly	Sometimes	Never
Clinical (positive findings on auscultation such as bronchi, crepitations, wheeze, breathlessness, purulent sputum, impaired oxygenation or ventilation)	○	○	○	○
Radiological (CXR or CT showing lobar infiltration/bronchogram, diffuse/patchy shadowing)	○	○	○	○
Sonological (USS showing consolidation/bronchogram)	○	○	○	○

24. For each category of diagnostic criteria in CAP , select the most appropriate option.

	Some or all must be present for diagnosis	Supportive for diagnosis but not essential	Not part of diagnostic assessment
Clinical (positive findings on auscultation such as bronchi, crepitations, wheeze, breathlessness, purulent sputum, impaired oxygenation or ventilation)	○	○	○
Radiological (CXR or CT showing lobar infiltration/bronchogram, diffuse/patchy shadowing)	○	○	○
Sonological (USS showing consolidation/bronchogram)	○	○	○

25. Regarding the microbiological diagnosis of pneumonia **CAP**, how often do you assess each of the following?

	Always	Mostly	Sometimes	Never
Sputum/Endotracheal apirate	○	○	○	○
Blind Mini-BAL (ventilated patients only)	○	○	○	○
Bronchoscopic BAL (ventilated patient)	○	○	○	○
Bronchoscopic BAL (spontaneously breathing patient)	○	○	○	○
Blood cultures	○	○	○	○
Other body fluids (eg pleural fluid)	○	○	○	○

26. What is your initial antibiotic regime for **CAP**?

- ○ Monotherapy
- ○ Dual therapy including a macrolide
- ○ Dual therapy including a non-macrolide

27. State the intended duration of therapy in **CAP** if patient responds to initial treatment

- ○ 4 days
- ○ 5-7 days
- ○ 8-12 days
- ○ >12 days
- ○ Other (please specify)

[ ]

28. In **Hospital-acquired pneumonia (HAP)**, how frequently is each of the following diagnostic criteria assessed? Select the most appropriate response:

	Always	Mostly	Sometimes	Never
Clinical (positive findings on auscultation such as bronchi, crepitations, wheeze, breathlessness, purulent sputum, impaired oxygenation or ventilation)	○	○	○	○
Radiological (CXR or CT showing lobar infiltration/bronchogram, diffuse/patchy shadowing)	○	○	○	○
Sonological (USS showing consolidation/bronchogram)	○	○	○	○

29. For each category of diagnostic criteria in **HAP** , select the most appropriate option.

	Some or all must be present for diagnosis	Supportive for diagnosis but not essential	Not part of diagnostic assessment
Clinical (positive findings on auscultation such as bronchi, crepitations, wheeze, breathlessness, purulent sputum, impaired oxygenation or ventilation)	○	○	○
Radiological (CXR or CT showing lobar infiltration/bronchogram, diffuse/patchy shadowing)	○	○	○
Sonological (USS showing consolidation/bronchogram)	○	○	○

30. Regarding the microbiological diagnosis of pneumonia HAP, how often do you assess each of the following?

	Always	Mostly	Sometimes	Never
Sputum/Endotracheal aspirate	○	○	○	○
Blind mini-BAL (ventilated patients only)	○	○	○	○
Bronchoscopic BAL (ventilated patients)	○	○	○	○
Bronchoscopic BAL (spontaneously breathing patients)	○	○	○	○
Blood cultures	○	○	○	○
Other body fluids (eg pleural fluid)	○	○	○	○

31. What is your initial antibiotic regime for **HAP**?

- ○ Monotherapy for all patients
- ○ Dual therapy including coverage for resistant organisms (eg MRSA/MDR Pseudomonas) for all patients
- ○ Monotherapy for low risk of resistant organisms, dual therapy for high risk of resistant organisms (eg MRSA/MDR pseudomonas)

32. State the intended duration of therapy in **HAP** if patient reponds to initial treatment:

- ○ 4 days
- ○ 5-7 days
- ○ 8-12 days
- ○ >12 days
- ○ Other (please specify):

[ ]

33. Do you use an ‘antibiotic time out’ to review continuing antibiotics at 48-72 hours in **HAP**?

- ◯ Yes
- ◯ No

34. In **VAP**, how frequently is each of the following diagnostic criteria assessed? Select the most appropriate response:

	Always	Mostly	Sometimes	Never
Clinical (positive findings on auscultation such as bronchi, crepitations, wheeze, breathlessness, purulent sputum, impaired oxygenation or ventilation)	◯	◯	◯	◯
Radiological (CXR or CT showing lobar infiltration/bronchogram, diffuse/patchy shadowing)	◯	◯	◯	◯
Sonological (USS showing consolidation/bronchogram)	◯	◯	◯	◯

35. For each category of diagnostic criteria in **VAP** , select the most appropriate option.

	Some or all must be present for diagnosis	Supportive for diagnosis but not essential	Not part of diagnostic assessment
Clinical (positive findings on auscultation such as bronchi, crepitations, wheeze, breathlessness, purulent sputum, impaired oxygenation or ventilation)	◯	◯	◯
Radiological (CXR or CT showing lobar infiltration/bronchogram, diffuse/patchy shadowing)	◯	◯	◯
Sonological (USS showing consolidation/bronchogram)	◯	◯	◯

36. Regarding the microbiological diagnosis of pneumonia **VAP**, how often do you assess each of the following?

	Always	Mostly	Sometimes	Never
Sputum/Endotracheal aspirate	◯	◯	◯	◯
Blind mini-BAL	◯	◯	◯	◯
Bronchoscopic BAL	◯	◯	◯	◯
Blood cultures	◯	◯	◯	◯
Other body fluids (e.g. pleural fluid)	◯	◯	◯	◯

37. What is your initial antibiotic regime for **VAP**?

- Monotherapy for all patients
- Dual therapy including coverage for resistant organisms (eg MRSA/MDR Pseudomonas) for all patients
- Monotherapy for low risk of resistant organisms, dual therapy for high risk of resistant organisms (eg MRSA/MDR pseudomonas)

38. State the intended duration of therapy in **VAP** if patient responds to initial treatment:

- 4 days
- 5-7 days
- 8-12 days
- >12 days
- Other (please specify):

39. Do you use an ‘antibiotic time out’ to review continuing antibiotics at 48-72 hours in **VAP**?

- Yes
- No
